# A Cavity‐Tailored Metal‐Organic Cage Entraps Gases Selectively in Solution and the Amorphous Solid State

**DOI:** 10.1002/anie.202102095

**Published:** 2021-05-04

**Authors:** Jun‐Long Zhu, Dawei Zhang, Tanya K. Ronson, Wenjing Wang, Lin Xu, Hai‐Bo Yang, Jonathan R. Nitschke

**Affiliations:** ^1^ Shanghai Key Laboratory of Green Chemistry and Chemical Processes School of Chemistry and Molecular Engineering East China Normal University 3663 N. Zhongshan Road Shanghai 200062 P. R. China; ^2^ Department of Chemistry University of Cambridge Lensfield Road Cambridge CB2 1EW UK; ^3^ State Key Laboratory of Structural Chemistry Fujian Institute of Research on the Structure of Matter Chinese Academy of Sciences Fuzhou 350002 China

**Keywords:** gas adsorption, gas encapsulation, host-guest chemistry, metal-organic cage, supramolecular chemistry

## Abstract

Here we report the subcomponent self‐assembly of a truxene‐faced Zn_4_L_4_ tetrahedron, which is capable of binding the smallest hydrocarbons in solution. By deliberately incorporating inward‐facing ethyl groups on the truxene faces, the resulting partially‐filled cage cavity was tailored to encapsulate methane, ethane, and ethene via van der Waals interactions at atmospheric pressure in acetonitrile, and also in the amorphous solid state. Interestingly, gas capture showed divergent selectivities in solution and the amorphous solid state. The selective binding may prove useful in designing new processes for the purification of methane and ethane as feedstocks for chemical synthesis.

## Graphical abstract

A Zn_4_L_4_ coordination cage enabled entrapment of gases in both solution and the amorphous solid state at atmospheric pressure. The lipophilic cavity of the cage was designed through a cavity‐filling strategy, which could bind small hydrocarbon gases. A contrasting selectivity of gas capture was observed in solution and the solid state, showing the potential of the cage to be engineered as porous liquids or solid adsorbents for gas separation.

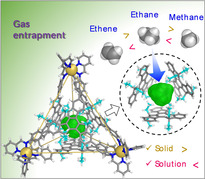

Hydrocarbon gases are as ubiquitous as they are industrially important. Methane is the least environmentally problematic hydrocarbon fuel, and also an essential raw material for industry.^[1]^ Ethene is also widely used in industry, primarily in the production of polyethylene. Ethene is produced primarily from naphtha or ethane, requiring its separation and purification from ethane.^[2]^ The ability to selectively bind these hydrocarbon gases is crucial for applications such as gas separation and storage.

Crystalline porous materials, such as metal‐organic frameworks (MOFs)^[3]^ or covalent‐organic frameworks (COFs),^[4]^ have been investigated as adsorbents for gas separation and storage.^[5]^ The crystal lattices of these materials may take up gases into large lattice voids or interstitial spaces. Discrete, soluble molecular containers^[6]^ offer properties that are complementary to those of crystalline framework materials. The encapsulation of gases in solution may allow for new purification strategies to be deployed, for instance by incorporating containers into separation films, or carrying out gas separation in solution under flow. The prospect of these applications has greatly stimulated the development of the emerging area of porous liquids.^[7]^ Gas encapsulation within soluble containers may also increase the solubility of gases in solution,^[7a]^ which may in turn enable these gases to become the substrates of cage‐catalyzed reactions.^[8]^

Soluble containers bind gases in different ways than do framework materials.^[9]^ The use of purely organic capsules^[10]^ for gas binding has been well explored,^[11]^ whereas gas encapsulation within discrete metal‐organic cages^[12]^ has been observed in a much more limited set of cases.^[13]^ A water‐soluble Fe^II^_4_L_6_ coordination cage was found to encapsulate SF_6_ or Xe in water via the hydrophobic effect.[^13a^, ^13b^] A Fe^II^_4_L_4_ tetrahedron that bound cryptophane‐111 formed a cage‐in‐cage host, which further bound Xe.^[13c]^ Recently, Li et al. presented the entrapment of CO_2_ by Ni‐imidazolate cages in solution at high CO_2_ pressure, which also occurred in the crystalline state of the cages.^[13d]^ The encapsulation of hydrocarbon gases by metal‐organic cages has not been observed yet, to the best of our knowledge.

Based upon our experience using triazatruxene‐containing cage subcomponents,[^13c^, ^14^] we sought to explore cages containing alkylated truxene moieties. The alkyl groups of these cages were designed to project into and partially fill the cage cavity. Crucially, the aliphatic character of these groups provides an internal environment distinct from that of other metal‐organic cages, which are most often lined with aromatic panels.[^12g^, ^15^] Such a cavity might thus bind aliphatic hydrocarbons well, following the principle of “like dissolves like”. This hypothesis led us to synthesize tetrahedron **1** (Figure 1), which was shown to be capable of entrapping small hydrocarbon gases in both solution and the solid state.

**Figure 1:**
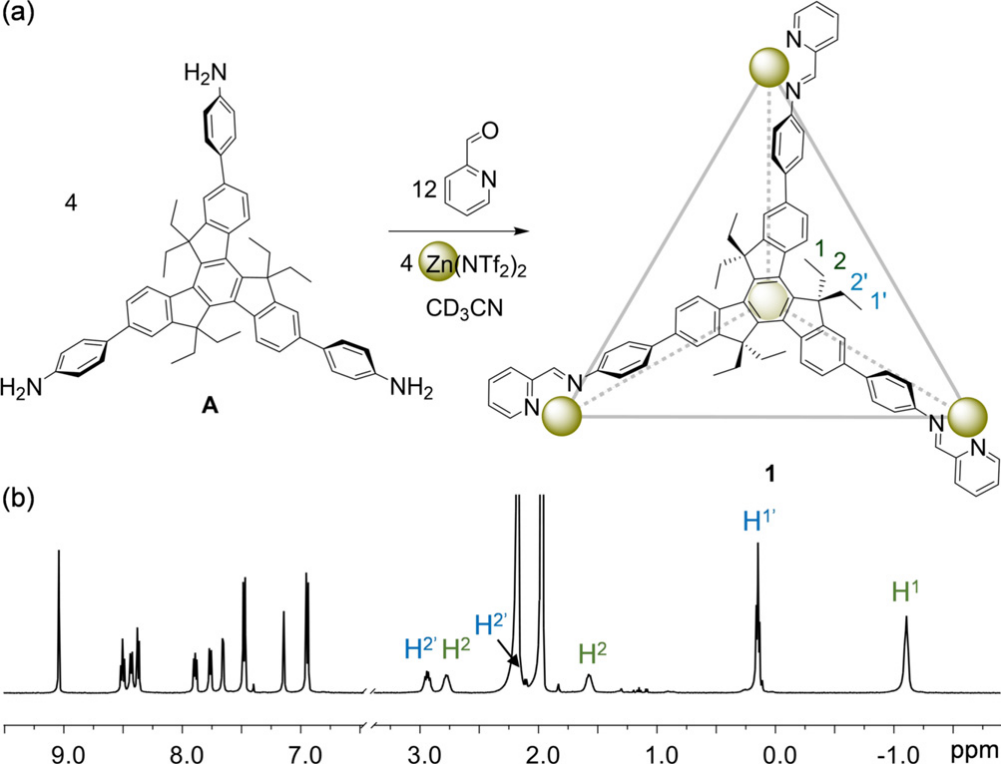
a) Subcomponent self‐assembly of tetrahedron **1** and b) its ^1^H NMR spectrum (CD_3_CN, 600 MHz, 298 K). The peaks of the two ethyl groups on each truxene are labelled.

Truxene‐based subcomponent **A** was obtained in four steps from commercially‐available starting materials (Scheme S1). The reaction of subcomponent **A** (4 equiv) with zinc(II) bis(trifluoromethane)sulfonimide (triflimide, Tf_2_N^−^, 4 equiv) and 2‐formylpyridine (12 equiv) in acetonitrile afforded tetrahedron **1** (Figures 1 and S4–S14). Its Zn_4_L_4_ composition was confirmed by ESI‐MS (Figure S11).

Although the clockwise/anticlockwise orientation of the truxene faces and the handedness of the tris‐chelated metal vertices of the tetrahedron might combine to allow for different diastereoisomers to form in solution,[^13c^, ^14^, ^16^] the ^1^H NMR spectrum of **1** displayed only one set of ligand signals (Figure 1 b), consistent with the exclusive formation of a pair of *T*‐symmetric tetrahedral enantiomers, having faces and vertices with a single stereochemical orientation.

The ethyl groups of **1** showed two sets of ^1^H NMR signals, with one set appearing at substantially lower chemical shift values (Figure 1 b). The peak at −1.1 ppm, assigned to the interior methyl groups, reflects the strong shielding experienced by these protons that are well ensconced inside the cavity.

Slow vapor diffusion of diethyl ether into an acetonitrile solution of **1** provided crystals suitable for X‐ray crystallographic analysis.^[17]^ As shown in Figure 2, four truxene ligands are observed to bridge four octahedral zinc(II) centers. Each ligand caps a face of the tetrahedron and displays a clockwise (C) or anticlockwise (A) orientation. Two enantiomers, A_4_Δ_4_‐**1** and C_4_Λ_4_‐**1**, are related by inversion in the unit cell. Half of the ethyl chains of the truxene moieties point into the cavity while the rest face outwards. Surrounding the inner cavity with ethyl chains results in a lipophilic confined volume of 86 Å^3^, calculated using VOIDOO.^[18]^ We envisaged this space to be suitable for the binding of small hydrocarbon gases via van der Waals interactions.[^11b^, ^11e^] Note that if 12 methylene groups were to be subtracted from or added into the cavity, the resulting cages bearing methyl or propyl substituents might be too large to bind gaseous guests or too crowded to form in solution.

**Figure 2:**
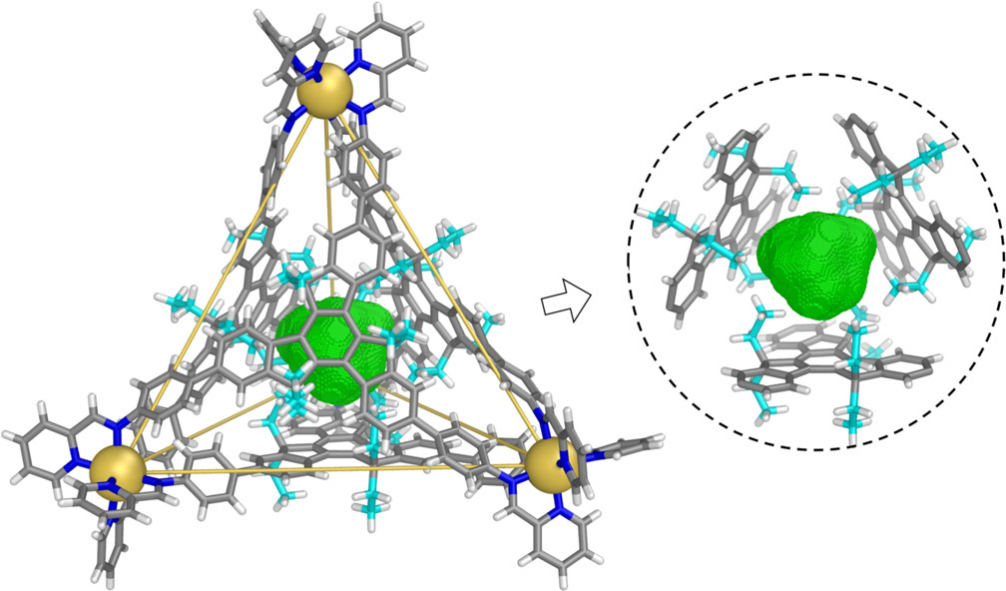
Crystal structure of tetrahedron **1** with its void space outlined in green mesh. The inset at right shows the cavity void surrounded by the ethyl substituents of the ligands. Ethyl carbon atoms are colored cyan. Disorder, counterions, and solvents of crystallization are omitted for clarity.

The encapsulation of the hydrocarbon gases methane, ethane, and ethene by tetrahedron **1** was investigated by bubbling these gases into an acetonitrile solution of **1** at 298 K followed by recording low‐temperature ^1^H NMR spectra. As shown in Figure 3 c, in addition to the single peak of free methane at 0.2 ppm, a new sharp peak at −2.6 ppm appeared at 238 K, indicating slow exchange guest binding on the NMR time scale. The upfield shift of the encapsulated methane signal is attributed to the strong shielding effect of the cage panels that surround it.

**Figure 3:**
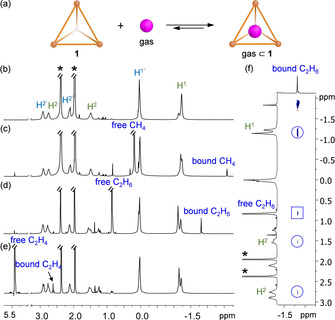
a) Encapsulation of the gases methane, ethane, and ethene within tetrahedron **1** in solution. Partial ^1^H NMR spectra (CD_3_CN, 600 MHz, 238 K) of b) free cage **1**; **1** binding c) methane, d) ethane, and e) ethene. f) ^1^H–^1^H NOESY spectrum (CD_3_CN, 600 MHz, 238 K) of **1** binding ethane. Cross‐peaks between encapsulated ethane and the ethyl groups of **1** are circled, and an exchange cross peak between the free and encapsulated ethane guest is highlighted by a square. Peaks from water and CHD_2_CN are indicated by asterisks.

Similarly, bubbling ethane or ethene through the cage solution also resulted in the appearance of an upfield‐shifted ^1^H signal, which was assigned to the encapsulated ethane or ethene (Figure 3). DOSY experiments indicated that the encapsulated gases displayed the same diffusion rates as the cage, which were slower than those of the free gases (Figures S18, S23, and S28).

The binding constants for all three gases were estimated to be smaller than 50 M^−1^ based on ^1^H NMR integration of the free and bound guest peaks; the differences in intensities between these peaks precluded accurate binding constant determination. Similar binding affinities were observed in a more polar solvent mixture of CD_3_OD/CD_3_CN (4/1, v/v) (Figures S19, S24, and S29). Nevertheless, we were able to investigate the binding hierarchy in solution through competitive binding experiments, which gave a moderate selectivity of methane > ethane > ethene (Figures S30–S32). This order does not correlate with the volume occupancy ratios, where methane, ethane, and ethene occupy 33 %, 52 %, and 47 % of the cavity of **1**, respectively.^[11d]^

We infer the ethyl chains surrounding the cavity of **1** to play an important role in enabling the binding of the smallest hydrocarbon guests in solution. These flexible chains offer the guests a van der Waals embrace, and also may deform readily in order to allow the gaseous guests to diffuse into the cavity.

Only the ^1^H NMR signals corresponding to the terminal CH_3_ groups of the ethyl moieties of **1** (H^1^ in green text in Figure 3) were observed to shift upon guest binding, whereas all other host proton signals exhibited no discernible changes. This observation is consistent with guest binding exclusively within the central cavity volume shown in Figure 2. NOESY experiments further confirmed gas binding within this central cavity (Figures 3 f, S16, S21, and S26). Importantly, no gas binding phenomena were observed in solution using our previously reported triazatruxene‐based cage^[14]^ (Figure S33), which had no inward‐facing ethyl groups, highlighting the essential role of these alkyl moieties for inducing gas binding.

We next examined gas binding within **1** in the solid state. Cage **1** was obtained as a powder following its precipitation from acetonitrile by adding diethyl ether. Thermogravimetric analysis (TGA) of **1** indicated stability to 400 °C (Figure S34) under N_2_. Cage **1** was thus activated by heating at 120 °C under vacuum (<0.013 mbar) for 10 h to remove solvent. Powder X‐ray diffraction (PXRD) of **1** revealed no distinct sharp peaks, consistent with the presence of amorphous material (Figure S35).

Gas adsorption isotherms of amorphous solid **1** for methane, ethane, and ethene at 295 K are shown in Figure 4. These isotherms display differing degrees of adsorption affinity at low pressures, in the order ethene > ethane ≫ methane. No obvious uptake of N_2_ or H_2_ was observed under the same conditions (<1 cm^3^ g^−1^, Figure S38). Although the actual adsorption selectivity remains to be determined from separation experiments on gas mixtures, this order based on the individual adsorption measurements contrasts with the selectivity observed in solution, which shows the strongest binding for methane. We infer these hydrocarbon gases to bind primarily within the cavity of **1**,[^13d^, ^19^] but some binding in the spaces between cages may also occur. We note that the adsorption of one molecule per cage corresponds to a theoretical uptake value of 3.6 cm^3^ g^−1^ for an ideal gas at 101 kPa and 295 K, which is the same order of magnitude as observed for these hydrocarbon gaseous guests.

**Figure 4:**
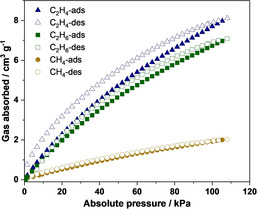
Gas adsorption isotherms of **1** for CH_4_ (circles), C_2_H_6_ (squares), and C_2_H_4_ (triangles) at 295 K.

High‐pressure gas adsorption experiments enable an increase in the gas adsorption capacity of **1**, for instance from 1.9 cm^3^ g^−1^ at 1 bar to 16 cm^3^ g^−1^ at 50 bar for methane (Figure S39). The presence of significant adsorption–desorption hysteresis indicates that once trapped, the guests are difficult to remove.

In conclusion, we have demonstrated a strategy for tailoring the cavity of a metal‐organic cage with simple aliphatic chains to enable the binding of small hydrocarbon gases. This strategy might be further elaborated to allow other functional capsules with varying cavity sizes to be constructed for more complex guest‐binding tasks.^[15a]^ Noting that Xe (van der Waals volume 42 Å^3^) has been bound previously,[^13b^, ^13c^] the smaller CH_4_ (28 Å^3^)[^11a^, ^11d^] for **1** in this case represents the smallest molecule to be encapsulated within metal‐organic cages as yet. The system described herein may serve as the foundation for hydrocarbon‐binding cage‐based porous liquids^[7a]^ or new fluid processes for the separation, purification, or storage of hydrocarbon gases.^[20]^

## Conflict of interest

The authors declare no conflict of interest.

## Supporting information

As a service to our authors and readers, this journal provides supporting information supplied by the authors. Such materials are peer reviewed and may be re‐organized for online delivery, but are not copy‐edited or typeset. Technical support issues arising from supporting information (other than missing files) should be addressed to the authors.

Supplementary

Supplementary

Supplementary
