# Supplementary material for: A Cavity‐Tailored Metal‐Organic Cage Entraps Gases Selectively in Solution and the Amorphous Solid State
Source: Angew Chem Int Ed Engl. 2021 May 4;60(21):11789–92. doi: 10.1002/anie.202102095 (PMC8251750; doi:10.1002/anie.202102095)

checkCIF/PLATON report

You have not supplied any structure factors. As a result the full set of tests cannot be run.

THIS REPORT IS FOR GUIDANCE ONLY. IF USED AS PART OF A REVIEW PROCEDURE FOR PUBLICATION, IT SHOULD NOT REPLACE THE EXPERTISE OF AN EXPERIENCED CRYSTALLOGRAPHIC REFEREE.

No syntax errors found. CIF dictionary Interpreting this report

Datablock: jnb223_sq

```
Bond precision:  C-C = 0.0160 A          Wavelength=1.54178

Cell:          a=21.6772(12)      b=28.3510(16)    c=37.125(2)
               alpha=102.949(2)   beta=95.153(3)   gamma=99.154(3)
Temperature:   180 K
```

	Calculated	Reported
Volume	21765(2)	21765(2)
Space group	P -1	P -1
Hall group	-P 1	-P 1
Moiety formula		
Sum formula	C313.99 H270 F29.96 N31 O19.97 S9.99 Zn4 [+ solvent]	C320 H270 F48 N34 O32 S16 Zn4
Mr	5947.78	6790.12
Dx,g cm-3	0.908	1.036
Z	2	2
Mu (mm-1)	1.190	1.591
F000	6160.1	6984.0
F000’	6172.99	
h,k,lmax	18,24,32	18,24,32
Nref	29968	28897
Tmin,Tmax	0.795,0.880	0.567,0.749
Tmin’	0.716	

```
Correction method= # Reported T Limits: Tmin=0.567 Tmax=0.749
AbsCorr = MULTI-SCAN

Data completeness= 0.964          Theta(max)= 42.093

R(reflections)= 0.1086( 19346)    wR2(reflections)= 0.3213( 28897)

S = 1.046                   Npar= 4033
```

The following ALERTS were generated. Each ALERT has the format
test-name_ALERT_alert-type_alert-level.
Click on the hyperlinks for more details of the test.

Alert level A

```
THETM01_ALERT_3_A  The value of sine(theta_max)/wavelength is less than 0.550
            Calculated sin(theta_max)/wavelength =    0.4348
```

Author Response: The crystals were weakly diffracting and few reflections at greater than 1.15 A resolution were observed hence the data were trimmed accordingly.

Alert level B

```
PLAT088_ALERT_3_B Poor Data / Parameter Ratio ....................       7.43 Note
```

Author Response: The low data to paramater ratio results from the limited resolution of the data.

```
PLAT341_ALERT_3_B Low Bond Precision on  C-C Bonds ..............    0.01602 Ang.
```

Author Response: The low bond precision arises from the limited resolution of the data and high levels of disorder within the anions.

Alert level C

```
PLAT029_ALERT_3_C _diffrn_measured_fraction_theta_full value Low .      0.964 Why?
PLAT082_ALERT_2_C High R1 Value ..................................       0.11 Report
PLAT084_ALERT_3_C High wR2 Value (i.e. > 0.25) ...................       0.32 Report
PLAT202_ALERT_3_C Isotropic non-H Atoms in Anion/Solvent .........          7 Check
              F1B     F2B     F3B     O1B     O2B     N1B                etc.
PLAT213_ALERT_2_C Atom N22             has ADP max/min Ratio .....        3.1 prolat
PLAT213_ALERT_2_C Atom C350            has ADP max/min Ratio .....        3.2 prolat
PLAT213_ALERT_2_C Atom C351            has ADP max/min Ratio .....        3.3 prolat
PLAT220_ALERT_2_C NonSolvent   Resd 1  C   Ueq(max)/Ueq(min) Range        5.8 Ratio
PLAT222_ALERT_3_C NonSolvent Resd 1  H   Uiso(max)/Uiso(min) Range        5.0 Ratio
PLAT241_ALERT_2_C High   'MainMol' Ueq as Compared to Neighbors of        C56 Check
PLAT241_ALERT_2_C High   'MainMol' Ueq as Compared to Neighbors of        C62 Check
PLAT241_ALERT_2_C High   'MainMol' Ueq as Compared to Neighbors of       C149 Check
PLAT241_ALERT_2_C High   'MainMol' Ueq as Compared to Neighbors of       C302 Check
PLAT241_ALERT_2_C High   'MainMol' Ueq as Compared to Neighbors of       C304 Check
PLAT241_ALERT_2_C High   'MainMol' Ueq as Compared to Neighbors of       C311 Check
PLAT241_ALERT_2_C High   'MainMol' Ueq as Compared to Neighbors of       C349 Check
PLAT241_ALERT_2_C High   'MainMol' Ueq as Compared to Neighbors of       C350 Check
PLAT241_ALERT_2_C High   'MainMol' Ueq as Compared to Neighbors of       C361 Check
PLAT242_ALERT_2_C Low    'MainMol' Ueq as Compared to Neighbors of        C70 Check
PLAT242_ALERT_2_C Low    'MainMol' Ueq as Compared to Neighbors of        C74 Check
PLAT242_ALERT_2_C Low    'MainMol' Ueq as Compared to Neighbors of       C174 Check
PLAT242_ALERT_2_C Low    'MainMol' Ueq as Compared to Neighbors of       C268 Check
PLAT242_ALERT_2_C Low    'MainMol' Ueq as Compared to Neighbors of       C368 Check
PLAT242_ALERT_2_C Low    'MainMol' Ueq as Compared to Neighbors of        S1F Check
PLAT244_ALERT_4_C Low    'Solvent' Ueq as Compared to Neighbors of        C1S Check
PLAT244_ALERT_4_C Low    'Solvent' Ueq as Compared to Neighbors of        C3S Check
```

```
PLAT250_ALERT_2_C Large U3/U1 Ratio for Average U(i,j) Tensor ....        2.4 Note
PLAT250_ALERT_2_C Large U3/U1 Ratio for Average U(i,j) Tensor ....        2.1 Note
PLAT260_ALERT_2_C Large Average Ueq of Residue Including       S1B      0.148 Check
PLAT260_ALERT_2_C Large Average Ueq of Residue Including       S1D      0.170 Check
PLAT260_ALERT_2_C Large Average Ueq of Residue Including       S1A      0.183 Check
PLAT260_ALERT_2_C Large Average Ueq of Residue Including       S1C      0.143 Check
PLAT260_ALERT_2_C Large Average Ueq of Residue Including       S1E      0.192 Check
PLAT260_ALERT_2_C Large Average Ueq of Residue Including       S1G      0.133 Check
PLAT260_ALERT_2_C Large Average Ueq of Residue Including       S1H      0.161 Check
PLAT260_ALERT_2_C Large Average Ueq of Residue Including       N1S      0.143 Check
PLAT260_ALERT_2_C Large Average Ueq of Residue Including       N2S      0.172 Check
PLAT410_ALERT_2_C Short Intra H...H Contact  H16B     ..H120     .       1.95 Ang.
                                                      x,y,z  =      1_555 Check
PLAT410_ALERT_2_C Short Intra H...H Contact  H20      ..H66A     .       1.99 Ang.
                                                      x,y,z  =      1_555 Check
PLAT410_ALERT_2_C Short Intra H...H Contact  H26F     ..H220     .       1.97 Ang.
                                                      x,y,z  =      1_555 Check
PLAT410_ALERT_2_C Short Intra H...H Contact  H37K     ..H338     .       1.98 Ang.
                                                      x,y,z  =      1_555 Check
PLAT733_ALERT_1_C Torsion Calc     0.2(18), Rep      0.1(4) ......       4.50 s.u.-R
              C151-N10 -C147-C148   1.555   1.555   1.555   1.555  #  393 Check
```

Alert level G

```
FORMU01_ALERT_1_G  There is a discrepancy between the atom counts in the
            _chemical_formula_sum and _chemical_formula_moiety. This is
            usually due to the moiety formula being in the wrong format.
            Atom count from _chemical_formula_sum:   C320 H270 F48 N34 O32 S16 Zn4
            Atom count from _chemical_formula_moiety:
FORMU01_ALERT_2_G  There is a discrepancy between the atom counts in the
            _chemical_formula_sum and the formula from the _atom_site* data.
            Atom count from _chemical_formula_sum:C320 H270 F48 N34 O32 S16 Zn4
            Atom count from the _atom_site data:  C313.9869 H270.0048 F29.95900 N3
CELLZ01_ALERT_1_G Difference between formula and atom_site contents detected.
CELLZ01_ALERT_1_G ALERT: Large difference may be due to a
            symmetry error - see SYMMG tests
           From the CIF: _cell_formula_units_Z    2
           From the CIF: _chemical_formula_sum  C320 H270 F48 N34 O32 S16 Zn4
           TEST: Compare cell contents of formula and atom_site data

           atom    Z*formula  cif sites diff
           C         640.00    627.97   12.03
           H         540.00    540.00    0.00
           F          96.00     59.92   36.08
           N          68.00     61.98    6.02
           O          64.00     39.94   24.06
           S          32.00     19.97   12.03
           Zn          8.00      8.00    0.00
PLAT002_ALERT_2_G Number of Distance or Angle Restraints on AtSite        467 Note
PLAT003_ALERT_2_G Number of Uiso or Uij Restrained non-H Atoms ...        473 Report
PLAT041_ALERT_1_G Calc. and Reported SumFormula    Strings  Differ     Please Check
PLAT042_ALERT_1_G Calc. and Reported MoietyFormula Strings  Differ     Please Check
PLAT051_ALERT_1_G Mu(calc) and Mu(CIF) Ratio Differs from 1.0 by .      25.18 %
PLAT068_ALERT_1_G Reported F000 Differs from Calcd (or Missing)...     Please Check
PLAT083_ALERT_2_G SHELXL Second Parameter in WGHT  Unusually Large     137.11 Why ?
PLAT172_ALERT_4_G The CIF-Embedded .res File Contains DFIX Records        131 Report
PLAT173_ALERT_4_G The CIF-Embedded .res File Contains DANG Records        233 Report
PLAT174_ALERT_4_G The CIF-Embedded .res File Contains FLAT Records          3 Report
PLAT175_ALERT_4_G The CIF-Embedded .res File Contains SAME Records          4 Report
PLAT178_ALERT_4_G The CIF-Embedded .res File Contains SIMU Records          4 Report
PLAT186_ALERT_4_G The CIF-Embedded .res File Contains ISOR Records          3 Report
```

```
PLAT187_ALERT_4_G The CIF-Embedded .res File Contains RIGU Records          1 Report
PLAT242_ALERT_2_G Low    'MainMol' Ueq as Compared to Neighbors of        C1F Check
PLAT242_ALERT_2_G Low    'MainMol' Ueq as Compared to Neighbors of        C2F Check
PLAT301_ALERT_3_G Main Residue  Disorder ..............(Resd  1  )         2% Note
PLAT302_ALERT_4_G Anion/Solvent/Minor-Residue Disorder (Resd  2  )       100% Note
PLAT302_ALERT_4_G Anion/Solvent/Minor-Residue Disorder (Resd  3  )       100% Note
PLAT302_ALERT_4_G Anion/Solvent/Minor-Residue Disorder (Resd  4  )       100% Note
PLAT302_ALERT_4_G Anion/Solvent/Minor-Residue Disorder (Resd  5  )       100% Note
PLAT302_ALERT_4_G Anion/Solvent/Minor-Residue Disorder (Resd  6  )       100% Note
PLAT302_ALERT_4_G Anion/Solvent/Minor-Residue Disorder (Resd  8  )       100% Note
PLAT302_ALERT_4_G Anion/Solvent/Minor-Residue Disorder (Resd  9  )       100% Note
PLAT304_ALERT_4_G Non-Integer Number of Atoms in ..... (Resd  2  )      12.13 Check
PLAT304_ALERT_4_G Non-Integer Number of Atoms in ..... (Resd  3  )      13.54 Check
PLAT304_ALERT_4_G Non-Integer Number of Atoms in ..... (Resd  4  )      10.23 Check
PLAT304_ALERT_4_G Non-Integer Number of Atoms in ..... (Resd  5  )       8.54 Check
PLAT304_ALERT_4_G Non-Integer Number of Atoms in ..... (Resd  6  )       7.84 Check
PLAT304_ALERT_4_G Non-Integer Number of Atoms in ..... (Resd  8  )       4.35 Check
PLAT304_ALERT_4_G Non-Integer Number of Atoms in ..... (Resd  9  )       3.27 Check
PLAT311_ALERT_2_G Isolated Disordered Oxygen Atom (No H's ?) .....        O1D Check
PLAT311_ALERT_2_G Isolated Disordered Oxygen Atom (No H's ?) .....        O2D Check
PLAT311_ALERT_2_G Isolated Disordered Oxygen Atom (No H's ?) .....        O3D Check
PLAT311_ALERT_2_G Isolated Disordered Oxygen Atom (No H's ?) .....        O4D Check
PLAT311_ALERT_2_G Isolated Disordered Oxygen Atom (No H's ?) .....        O1I Check
PLAT311_ALERT_2_G Isolated Disordered Oxygen Atom (No H's ?) .....        O2I Check
PLAT311_ALERT_2_G Isolated Disordered Oxygen Atom (No H's ?) .....        O3I Check
PLAT311_ALERT_2_G Isolated Disordered Oxygen Atom (No H's ?) .....        O4I Check
PLAT432_ALERT_2_G Short Inter X...Y Contact  S1E      ..C454           2.77 Ang.
                                                      x,y,z  =      1_555 Check
PLAT432_ALERT_2_G Short Inter X...Y Contact  S1E      ..C453           2.97 Ang.
                                                      x,y,z  =      1_555 Check
PLAT432_ALERT_2_G Short Inter X...Y Contact  O1E      ..C453           2.29 Ang.
                                                      x,y,z  =      1_555 Check
PLAT432_ALERT_2_G Short Inter X...Y Contact  O1E      ..C454           2.73 Ang.
                                                      x,y,z  =      1_555 Check
PLAT432_ALERT_2_G Short Inter X...Y Contact  O2E      ..C454           2.26 Ang.
                                                      x,y,z  =      1_555 Check
PLAT432_ALERT_2_G Short Inter X...Y Contact  O2E      ..C358           2.92 Ang.
                                                      x,y,z  =      1_555 Check
PLAT432_ALERT_2_G Short Inter X...Y Contact  O4E      ..C350           2.98 Ang.
                                                 1-x,1-y,-z  =      2_665 Check
PLAT606_ALERT_4_G Solvent Accessible VOID(S) in Structure ........          ! Info
PLAT720_ALERT_4_G Number of Unusual/Non-Standard Labels ..........          6 Note
PLAT794_ALERT_5_G Tentative Bond Valency for Zn1       (II)      .       1.67 Info
PLAT794_ALERT_5_G Tentative Bond Valency for Zn3       (II)      .       1.72 Info
PLAT794_ALERT_5_G Tentative Bond Valency for Zn4       (II)      .       1.69 Info
PLAT860_ALERT_3_G Number of Least-Squares Restraints .............       9180 Note
PLAT869_ALERT_4_G ALERTS Related to the Use of SQUEEZE Suppressed           ! Info
PLAT883_ALERT_1_G No Info/Value for _atom_sites_solution_primary .     Please Do !
PLAT933_ALERT_2_G Number of OMIT Records in Embedded .res File ...         15 Note
PLAT941_ALERT_3_G Average HKL Measurement Multiplicity ...........        2.3 Low
PLAT965_ALERT_2_G The SHELXL WEIGHT Optimisation has not Converged     Please Check
```

---

1 **ALERT level A** = Most likely a serious problem - resolve or explain
2 **ALERT level B** = A potentially serious problem, consider carefully
42 **ALERT level C** = Check. Ensure it is not caused by an omission or oversight
61 **ALERT level G** = General information/check it is not something unexpected

9 ALERT type 1 CIF construction/syntax error, inconsistent or missing data

```
58 ALERT type 2 Indicator that the structure model may be wrong or deficient
10 ALERT type 3 Indicator that the structure quality may be low
26 ALERT type 4 Improvement, methodology, query or suggestion
 3 ALERT type 5 Informative message, check
```

It is advisable to attempt to resolve as many as possible of the alerts in all categories. Often the minor alerts point to easily fixed oversights, errors and omissions in your CIF or refinement strategy, so attention to these fine details can be worthwhile. In order to resolve some of the more serious problems it may be necessary to carry out additional measurements or structure refinements. However, the purpose of your study may justify the reported deviations and the more serious of these should normally be commented upon in the discussion or experimental section of a paper or in the "special_details" fields of the CIF. checkCIF was carefully designed to identify outliers and unusual parameters, but every test has its limitations and alerts that are not important in a particular case may appear. Conversely, the absence of alerts does not guarantee there are no aspects of the results needing attention. It is up to the individual to critically assess their own results and, if necessary, seek expert advice.

Publication of your CIF in IUCr journals

A basic structural check has been run on your CIF. These basic checks will be run on all CIFs submitted for publication in IUCr journals (*Acta Crystallographica*, *Journal of Applied Crystallography*, *Journal of Synchrotron Radiation*); however, if you intend to submit to *Acta Crystallographica Section C* or *E* or *IUCrData*, you should make sure that full publication checks are run on the final version of your CIF prior to submission.

Publication of your CIF in other journals

Please refer to the *Notes for Authors* of the relevant journal for any special instructions relating to CIF submission.

PLATON version of 05/12/2020; check.def file version of 05/12/2020

Datablock jnb223_sq - ellipsoid plot

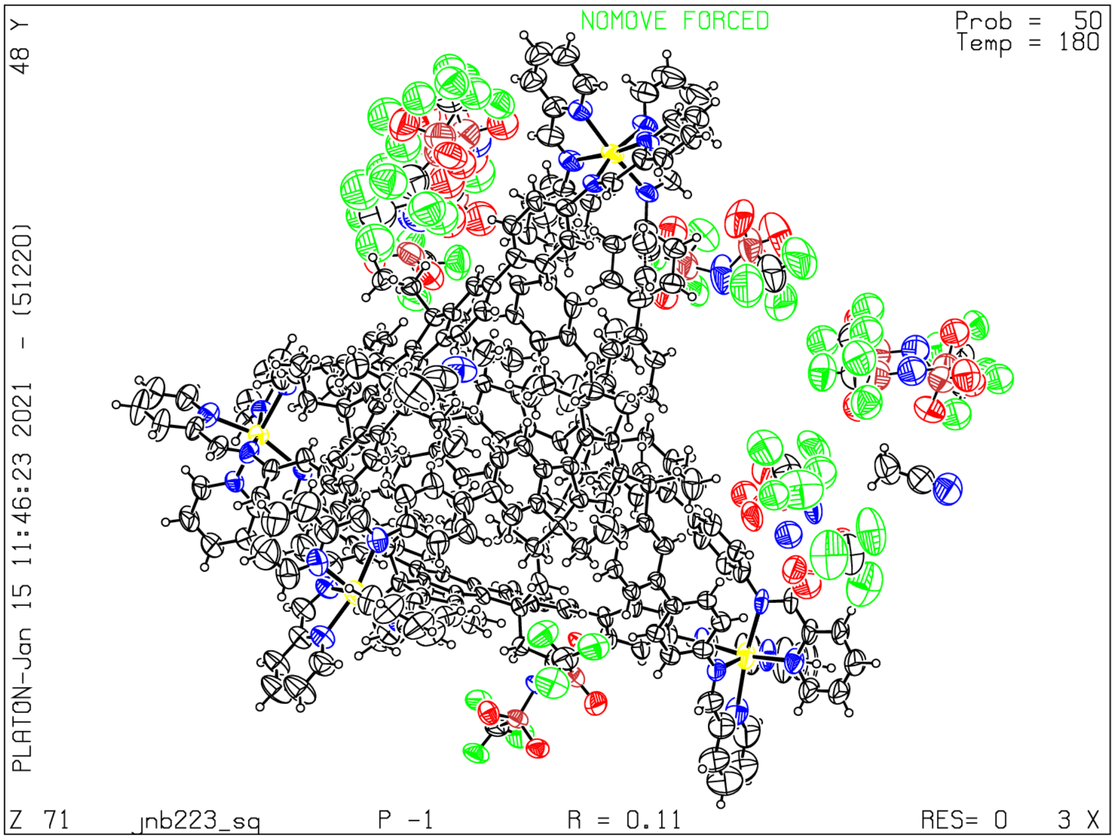

Supplement: Supplementary file 1 — Supplementary [file ANIE-60-11789-s001.pdf]
