# Supplementary material for: A Cavity‐Tailored Metal‐Organic Cage Entraps Gases Selectively in Solution and the Amorphous Solid State
Source: Angew Chem Int Ed Engl. 2021 May 4;60(21):11789–92. doi: 10.1002/anie.202102095 (PMC8251750; doi:10.1002/anie.202102095)
Supplement: Supplementary file 3 — Supplementary [file ANIE-60-11789-s003.pdf]

Angewandte Chemie
Eine Zeitschrift der Gesellschaft Deutscher Chemiker

Supporting Information

A Cavity-Tailored Metal-Organic Cage Entraps Gases Selectively in Solution and the Amorphous Solid State

Jun-Long Zhu[+], Dawei Zhang[+], Tanya K. Ronson, Wenjing Wang, Lin Xu, Hai-Bo Yang,* and Jonathan R. Nitschke**

anie_202102095_sm_miscellaneous_information.pdf

Contents

1. Materials and instrumentation 2

2. Synthesis and characterization 3

2.1 Synthesis of subcomponent **A** 3

2.2 Preparation and characterization of tetrahedron **1** 5

3. X-ray crystallography 12

4. Volume calculations 15

5. Gas binding studies of tetrahedron **1** in solution 16

5.1 Binding of CH_4 16

5.2 Binding of C_2H_6 19

5.3 Binding of C_2H_4 22

5.4 Competitive gas binding studies 25

6. Characterization of amorphous solid **1** 28

7. Gas adsorption properties of solid **1** 30

8. References 31

1. Materials and instrumentation

Unless otherwise specified, reagents and solvents were purchased from commercial suppliers and used without further purification.

Centrifugation of cage samples was carried out using a Grant-Bio LMC-3000 low speed benchtop centrifuge.

NMR spectra were recorded on a Bruker 300 MHz spectrometer, a Bruker 400 MHz spectrometer, a Bruker 500 MHz spectrometer and a Bruker 600 MHz spectrometer. Chemical shifts (δ) for ^{1}H NMR and ^{13}C NMR spectra are reported in parts per million (ppm) and are reported relative to the solvent residual peak (CD_3CN, $\delta_H = 1.94$ ppm, $\delta_C = 1.32$ ppm). Chemical shifts (δ) for ^{19}F NMR spectra are reported relative to hexafluorobenzene at -164.9 ppm. Coupling constants (*J*) are reported in hertz (Hz). All measurements were carried out at 298 K unless reported otherwise. The following abbreviations are used to describe signal multiplicity for ^{1}H, ^{13}C and ^{19}F NMR spectra: s: singlet, d: doublet, t: triplet, dd: doublet of doublets; dt: doublet of triplets; m: multiplet.

Low resolution electrospray ionisation mass spectrometry (ESI-MS) was carried out on a Micromass Quattro LC. The HR-ESI mass spectra were performed on an Agilent (Santa Clara, CA, USA) ESI-TOF mass spectrometer (6224).

Thermogravimetric analyses (TGA) were carried out on a Perkin Elmer STA8000 thermal analyzer under a nitrogen atmosphere with a heating rate of 10 °C·min^{-1}.

The Powder X-ray diffraction (PXRD) patterns were collected on a Rigaku Ultima IV diffractometer using Cu Kα radiation at 35 kV and 25 mA in the 2θ angle range of 3-50° using a step size of 0.02° and at a scanning speed of 10° min^{-1}. The samples were prepared by filling the holder with the dry powder.

Scanning electron microscopy (SEM) images of the solutions were obtained using an S-4800 (Hitachi Ltd.) with an accelerating voltage of 3.0-10.0 kV.

All the low-pressure gas adsorption-desorption measurements were carried out by using an automatic volumetric adsorption equipment (Micromeritics, ASAP2020) at 295 K. Prior to the measurements, the samples were degassed at 120 °C under dynamic vacuum (below 10 μmHg) for 10 h to remove the adsorbed impurities.

High-pressure CH_4 adsorption isotherm measurements were carried out on an HPVA II high pressure volumetric analyzer at room temperature. Prior to gas adsorption measurements, the samples were degassed for 10 h at 120 °C.

2. Synthesis and characterization

2.1 Synthesis of subcomponent A

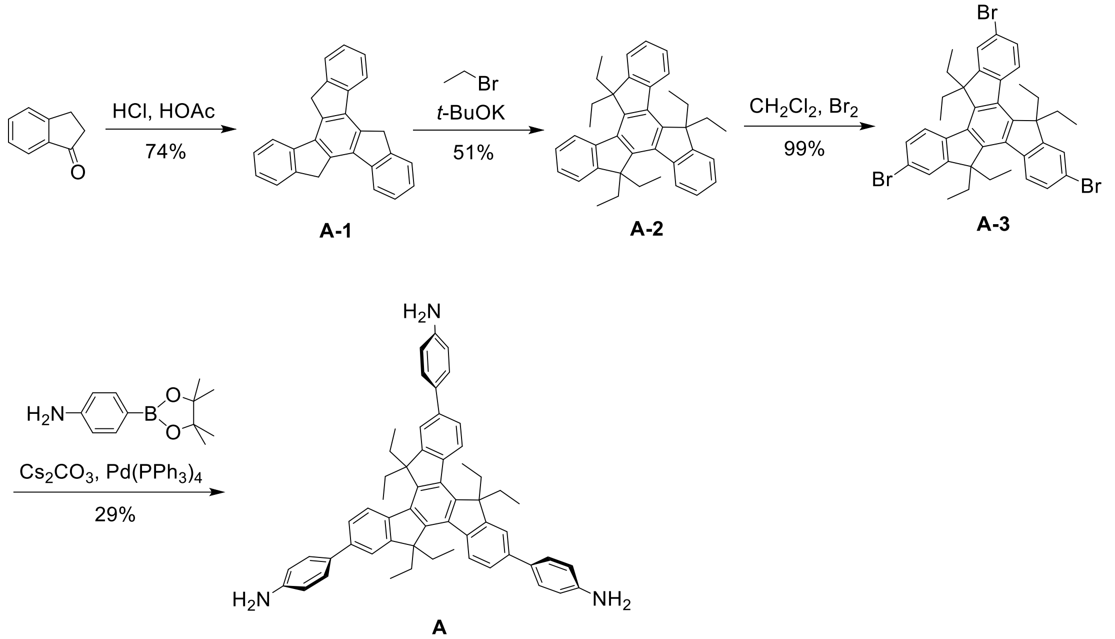

Scheme S1. Synthesis of subcomponent **A**.

Compounds **A-1**, **A-2**, and **A-3** were synthesized according to published procedures.[1]

A suspension of compound **A-3** (1.51 g, 2.01 mmol), 4-aminophenylboronic acid pinacol ester (3.04 g, 13.9 mmol), Cs_2CO_3 (4.00 g, 12.3 mmol) and $Pd(PPh_3)_4$ (622 mg, 0.538 mmol) in DMF/H_2O (45 mL/15 mL) was heated at 110 °C for 24 hours under nitrogen. Then the mixture was cooled to room temperature, diluted with water (100 mL) and extracted with CH_2Cl_2 (200 mL × 3). The combined organic phase was washed with brine (200 mL × 3), dried over Na_2SO_4 and the solvent was evaporated. The crude product was purified on a silica gel column using CH_2Cl_2 as the eluent to obtain pale yellow solid **A** (527 mg, 29%). **^{1}H NMR** (300 MHz, $CDCl_3$): δ 8.37 (d, *J* = 8.3 Hz, 3H), 7.64 – 7.53 (m, 12H), 6.83 (d, *J* = 8.4 Hz, 6H), 3.78 (s, 6H), 3.06 (dd, *J* = 13.4, 7.1 Hz, 6H), 2.21 (dd, *J* = 13.7, 7.2 Hz, 6H), 0.27 (t, *J* = 7.1 Hz, 18H) ppm. **^{13}C NMR** (125 MHz, $CDCl_3$): δ 153.5, 145.8, 143.7, 139.3, 139.2, 138.6, 131.7, 128.0, 124.8, 124.4, 120.0, 115.5, 56.8, 29.6, 8.7 ppm. **ESI-HRMS**: *m/z* Calcd for $C_{57}H_{58}N_3$: 784.4625. Found: 784.4619.

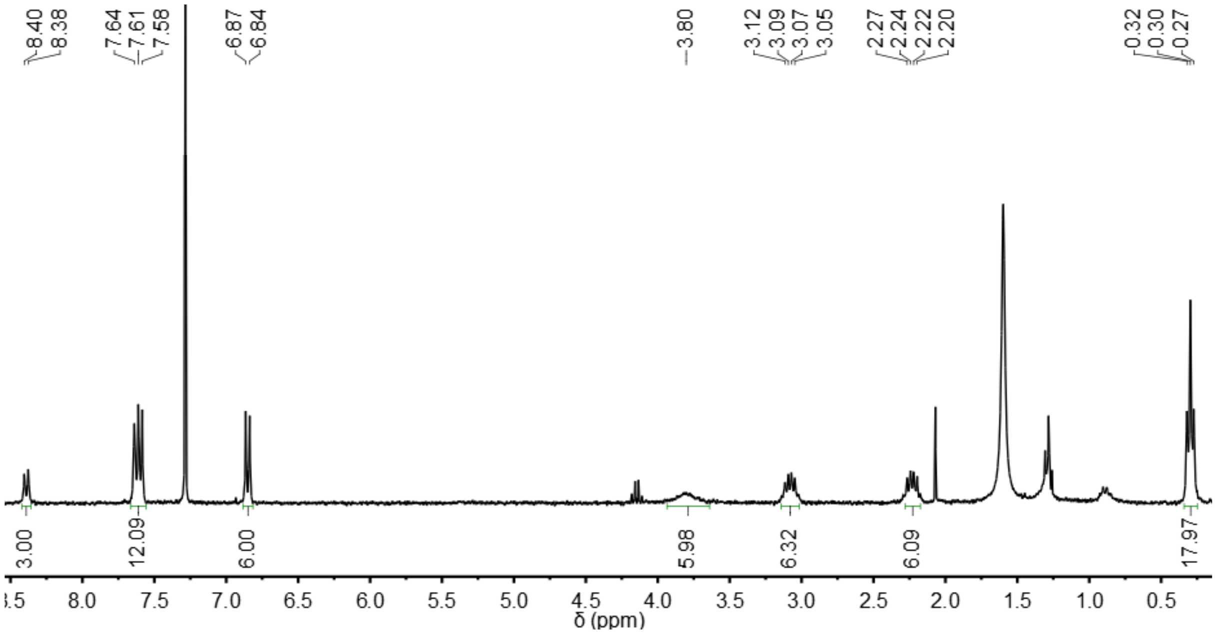

Figure S1. ^{1}H NMR spectrum of subcomponent **A** (300 MHz, $CDCl_3$, 298 K).

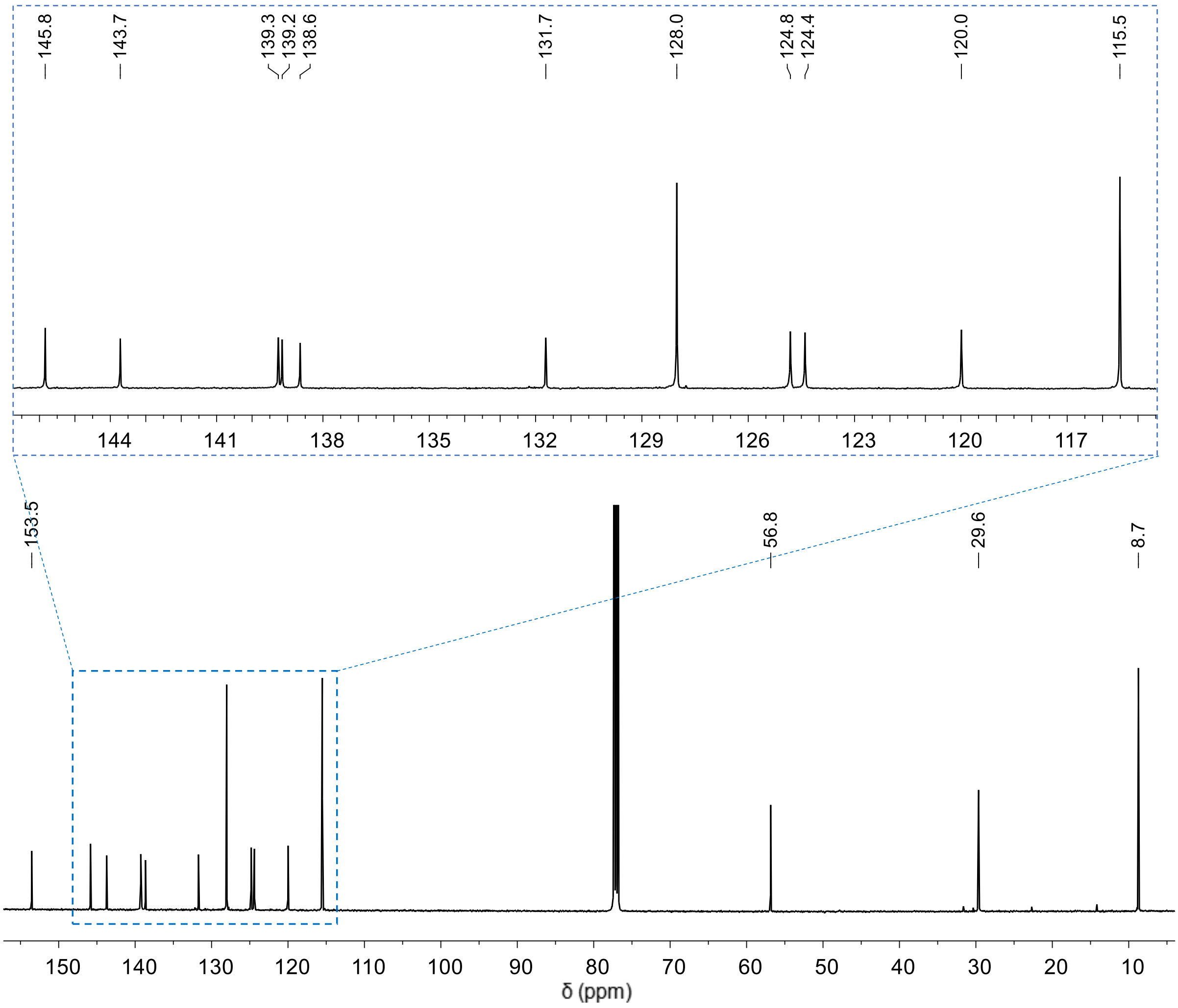

Figure S2. ^{13}C NMR spectrum of subcomponent **A** (125 MHz, $CDCl_3$, 298 K).

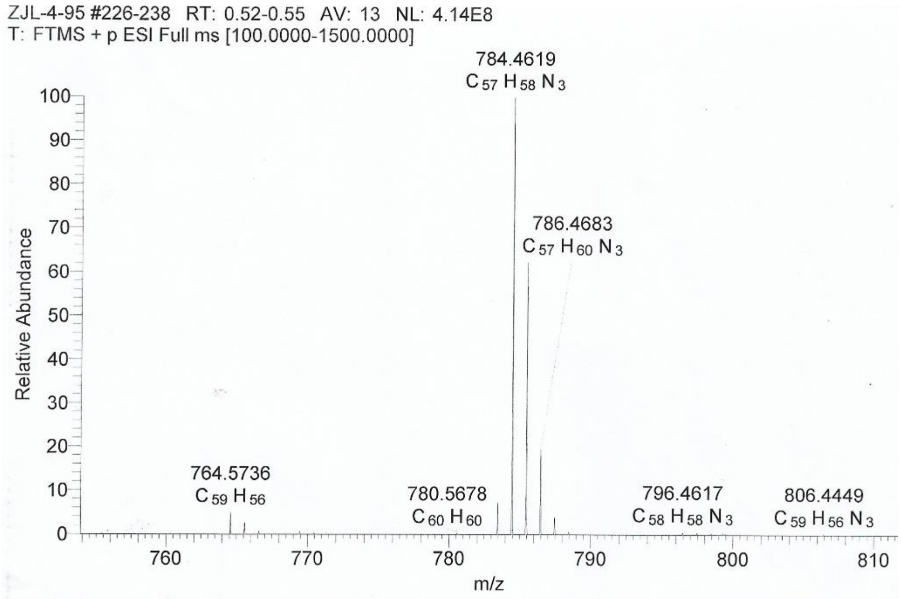

ZJL-4-95#226-238 RT: 0.52-0.55 AV: 13
T: FTMS + p ESI Full ms [100.0000-1500.0000]
m/z= 753.8772-811.8944

m/z	Intensity	Relative	Theo. Mass	Delta (ppm)	RDB equiv.	Composition
764.5736	20594392.0	4.94	764.4377	136.00	32.0	$C_{59}H_{56}$
765.5771	10813732.0	2.60	765.4455	131.61	31.5	$C_{59}H_{57}$
766.5807	2870638.0	0.69	766.4533	127.40	31.0	$C_{59}H_{58}$
769.4498	2644911.0	0.63	769.4516	-1.82	30.5	$C_{57}H_{57}N_2$
783.4540	30124914.0	7.23	783.4547	-0.66	31.0	$C_{57}H_{57}N_3$
784.4619	416687008.0	100.00	784.4625	-0.59	30.5	$C_{57}H_{58}N_3$
785.4650	258575072.0	62.05	785.4704	-5.32	30.0	$C_{57}H_{59}N_3$
786.4683	80388064.0	19.29	786.4782	-9.84	29.5	$C_{57}H_{60}N_3$
787.4718	16687305.0	4.00				
796.4617	2778790.5	0.67	796.4625	-0.80	31.5	$C_{58}H_{58}N_3$

Figure S3. High-resolution ESI-mass spectrum of subcomponent **A**.

2.2 Preparation and characterization of tetrahedron 1

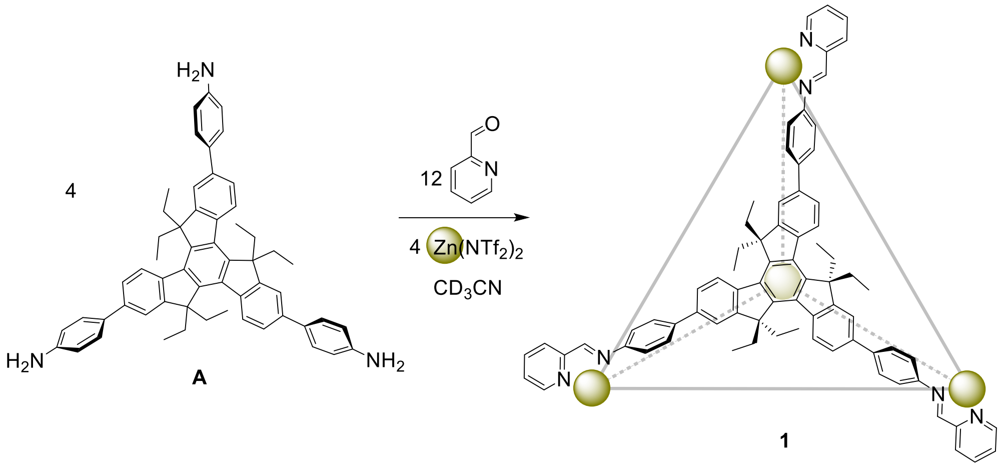

Scheme S2. Subcomponent self-assembly of tetrahedron **1**.

Subcomponent **A** (87.2 mg, 0.111 mmol), 2-pyridinecarboxaldehyde (106.09 mg, 0.58 mmol), and $Zn(NTf_2)_2$ (69.6 mg, 0.111 mmol) were combined in CH_3CN (10 mL) and stirred

at 70 °C overnight. The solvent was evaporated and diethyl ether (10 mL) was then added. The residue was resuspended and then centrifuged, and the diethyl ether was decanted. This procedure was repeated three times with fresh diethyl ether. The residue was then dried in vacuo to afford the solid product as orange powder (167 mg, 89%). **^{1}H NMR** (400 MHz, CD_3CN): δ 9.02 (s, 12H), 8.48 (td, J = 7.8, 1.4 Hz, 12H), 8.40 (d, J = 8.6 Hz, 12H), 8.34 (d, J = 7.4 Hz, 12H), 7.89 – 7.83 (m, 12H), 7.74 (d, J = 8.2 Hz, 12H), 7.63 (d, J = 5.2 Hz, 12H), 7.45 (d, J = 8.5 Hz, 24H), 7.12 (s, 12H), 6.92 (d, J = 8.5 Hz, 24H), 2.94 – 2.88 (m, 12H), 2.78 – 2.72 (m, 12H), 2.17 – 2.10 (m, 12H), 1.57 – 1.51 (m, 12H), 0.12 (t, J = 7.2 Hz, 36H), -1.13 (t, J = 6.0 Hz, 36H). **^{13}C NMR** (125 MHz, CD_3CN): δ 163.2, 153.1, 149.1, 146.7, 145.4, 144.2, 142.5, 142.4, 139.9, 138.6, 138.2, 131.2, 130.6, 128.3, 125.4, 125.1, 123.8, 121.3, 120.0 (q, J = 325 Hz), 56.6, 29.7, 29.0, 7.9, 7.4 ppm. **^{19}F NMR** (376 MHz, CD_3CN, referenced to C_6F_6): δ (ppm) -80.62. **ESI-MS**: m/z 557.7 $[M]^{8+}$, 677.3 $[M+1Tf_2N^-]^{7+}$, 836.9 $[M+2Tf_2N^-]^{6+}$, 1060.2 $[M+3Tf_2N^-]^{5+}$, 1395.3 $[M+4Tf_2N^-]^{4+}$.

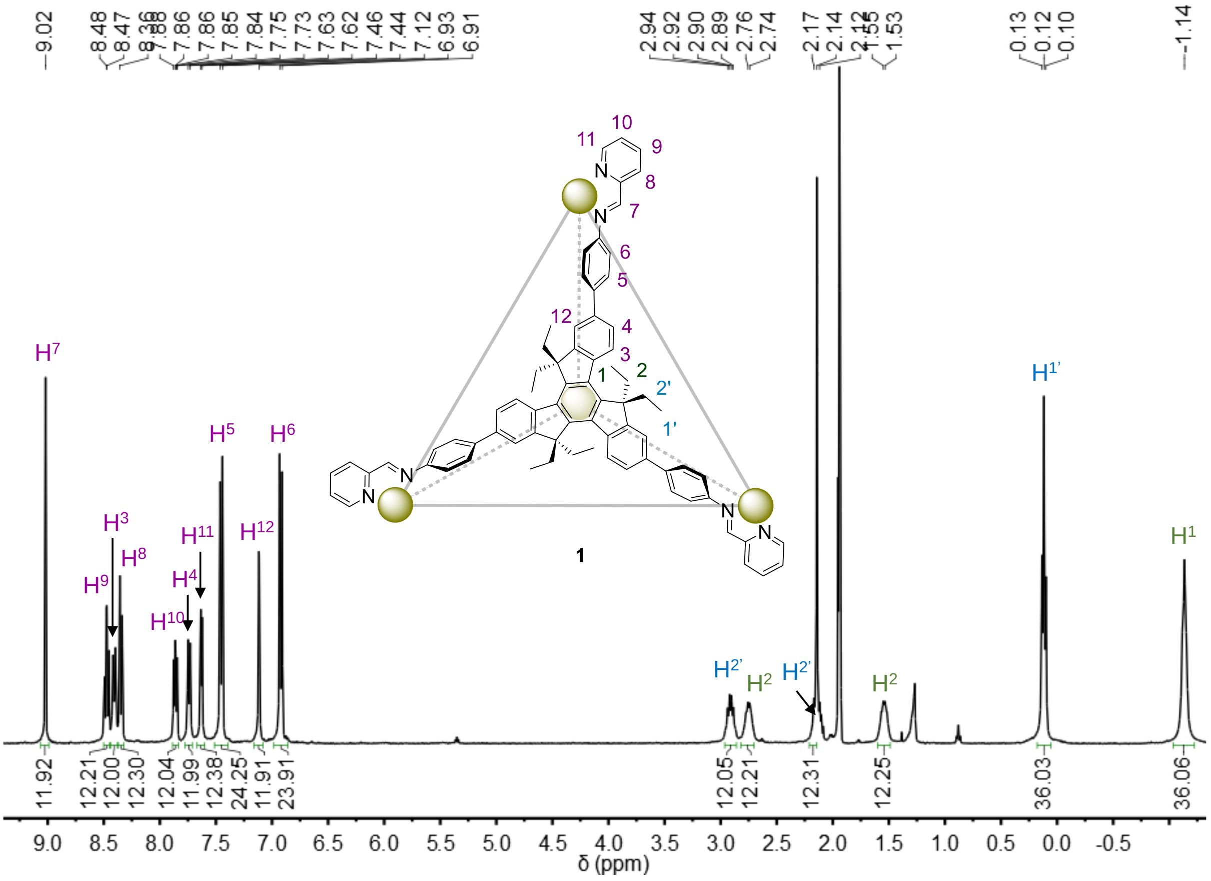

Figure S4. ^{1}H NMR spectrum of tetrahedron **1** (500 MHz, CD_3CN, 298 K).

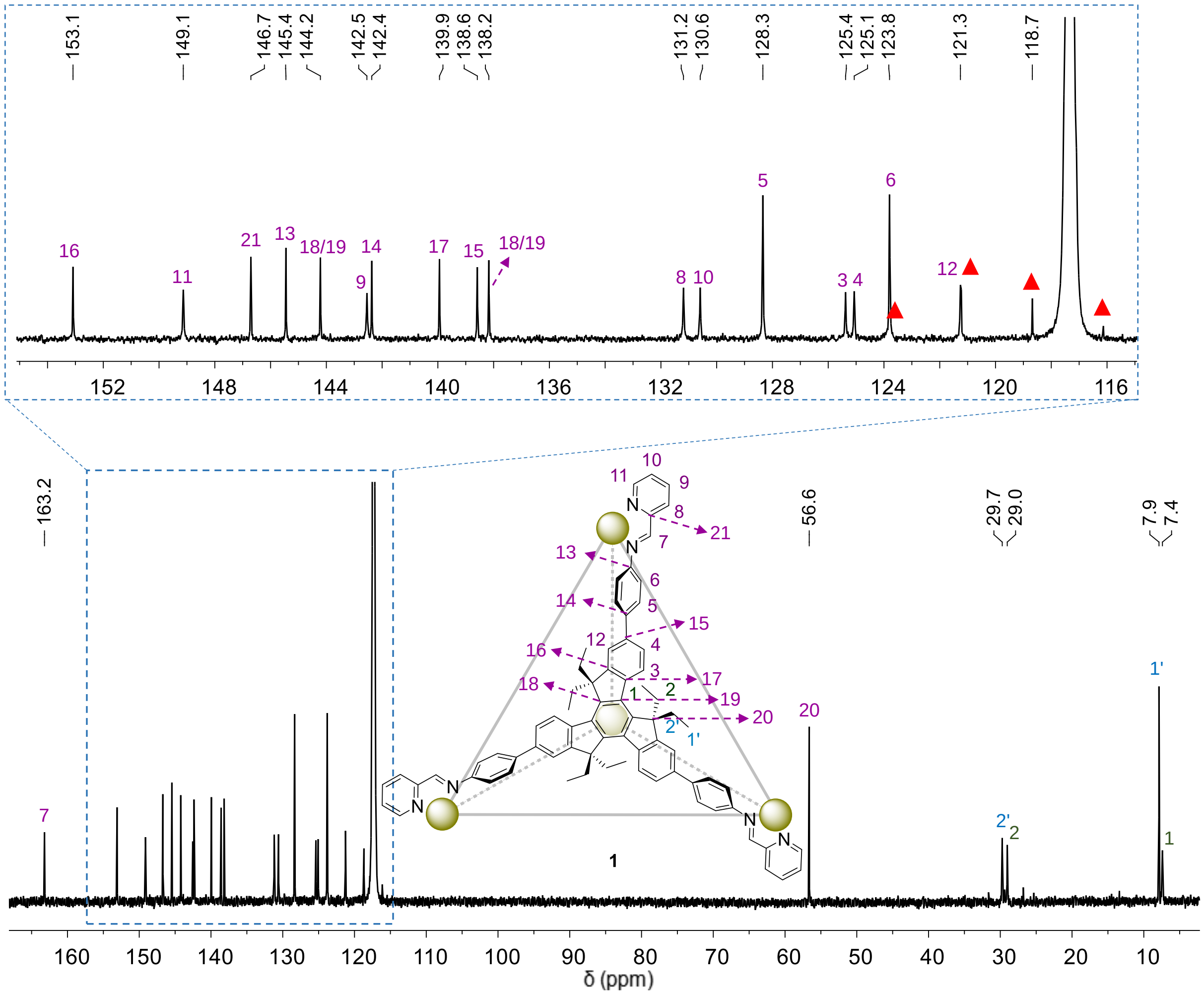

Figure S5. ^{13}C NMR spectrum of tetrahedron **1** (125 MHz, CD_3CN, 298 k). The peaks resulting from Tf_2N^- have been labelled with ▲.

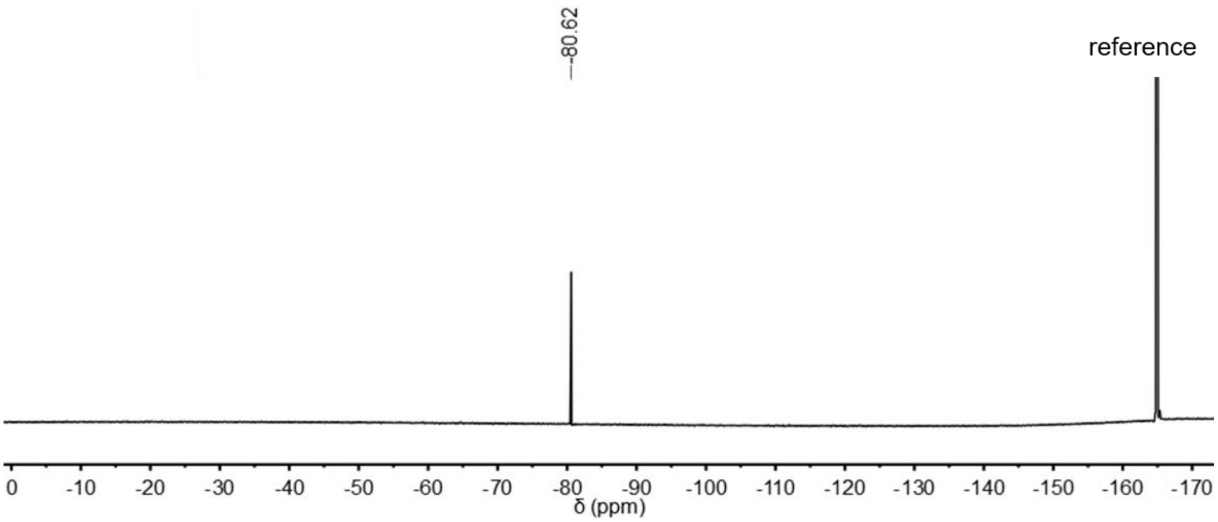

Figure S6. ^{19}F NMR spectrum of tetrahedron **1** (376 MHz, CD_3CN, 298 K, referenced to C_6F_6).

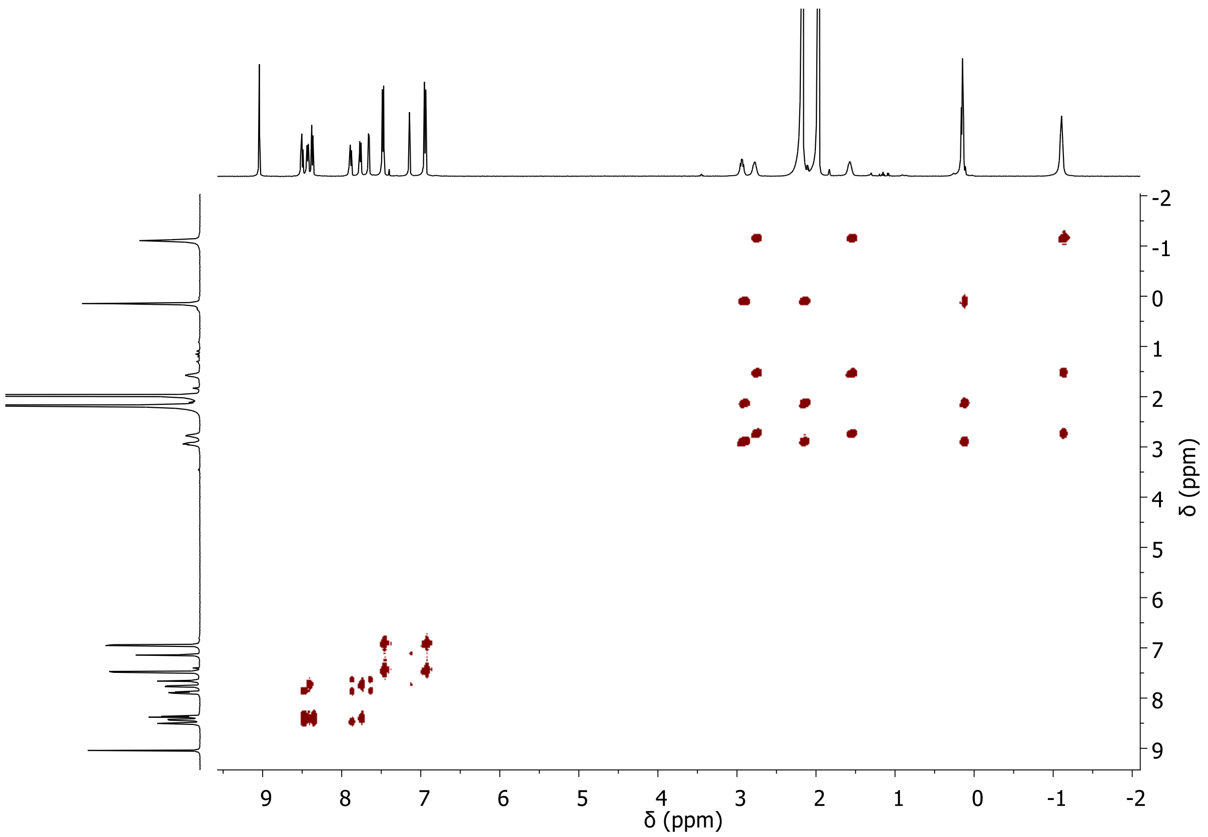

Figure S7. ^{1}H-^{1}H COSY spectrum of tetrahedron **1** (500 MHz, CD_3CN, 298 K).

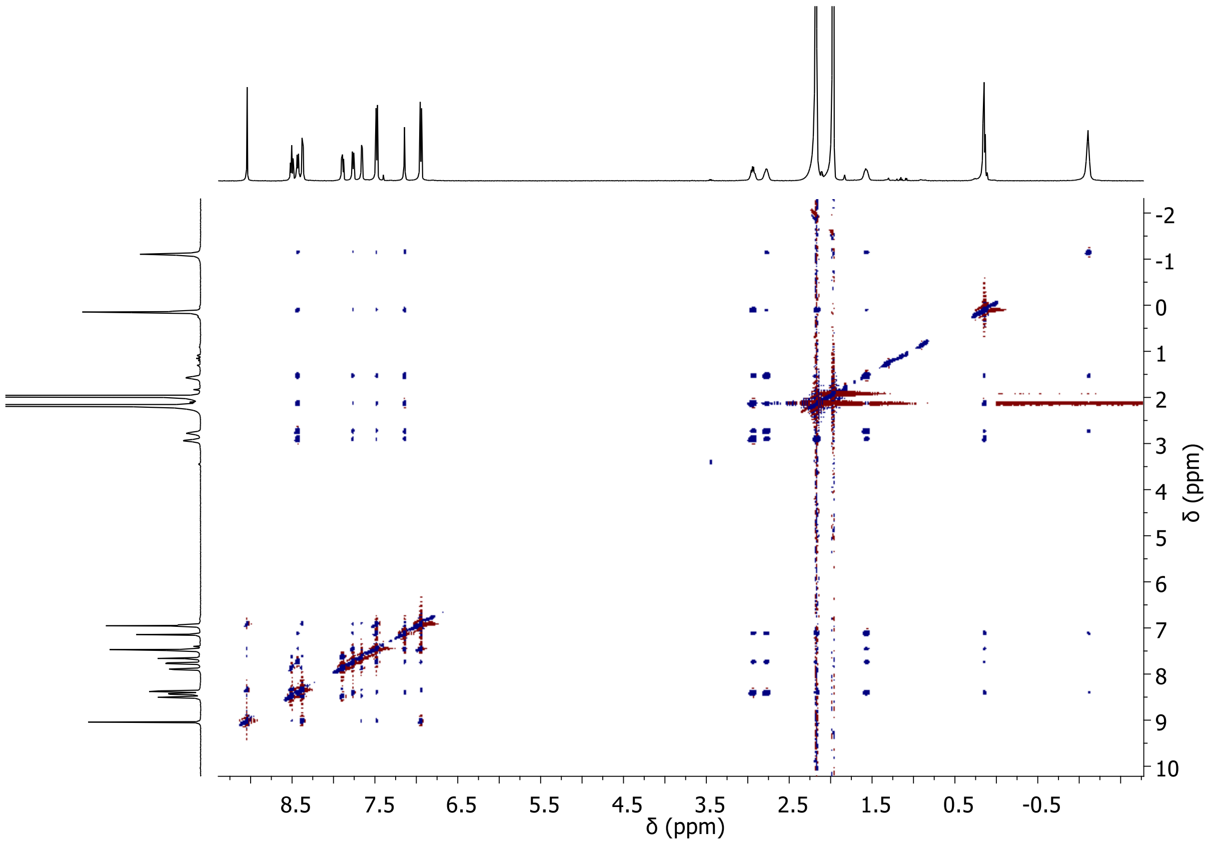

Figure S8. ^{1}H-^{1}H NOESY spectrum of tetrahedron **1** (500 MHz, CD_3CN, 298 K).

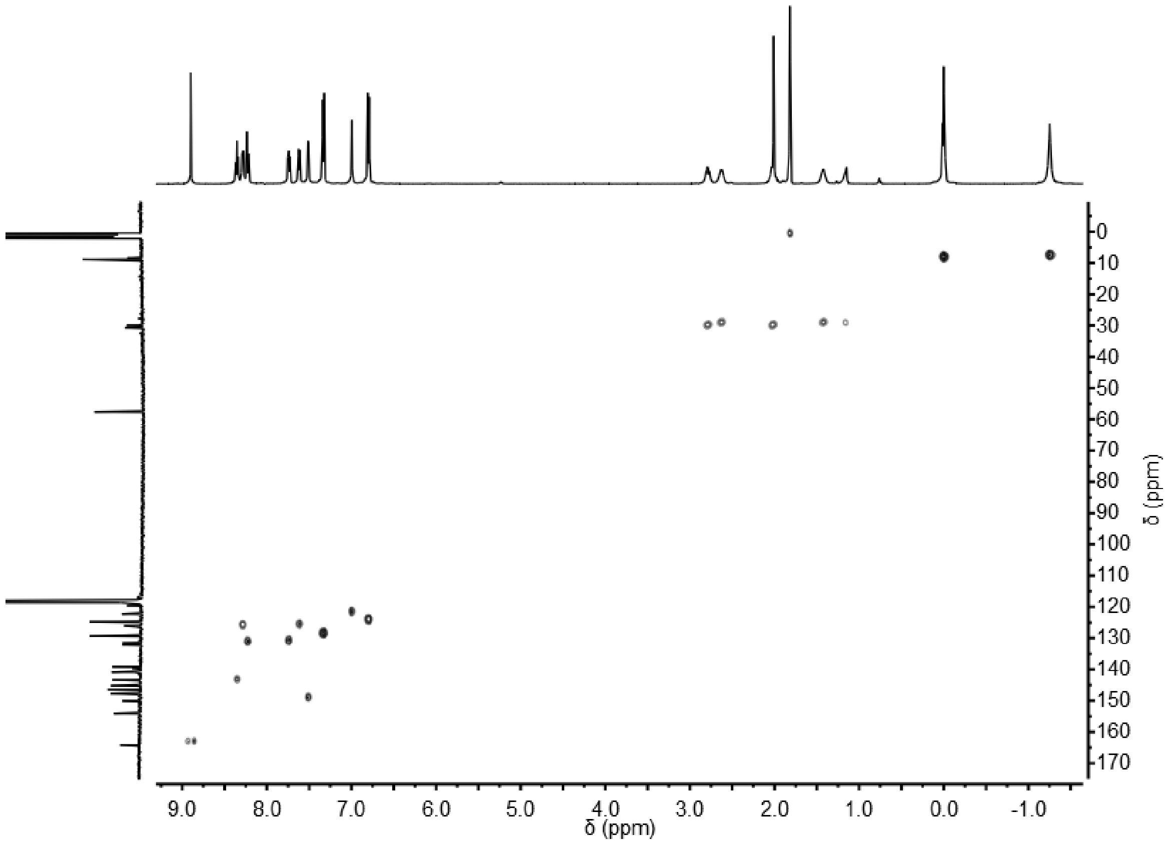

Figure S9. ^{1}H-^{13}C HSQC spectrum of tetrahedron **1** (500 MHz, CD_3CN, 298 K).

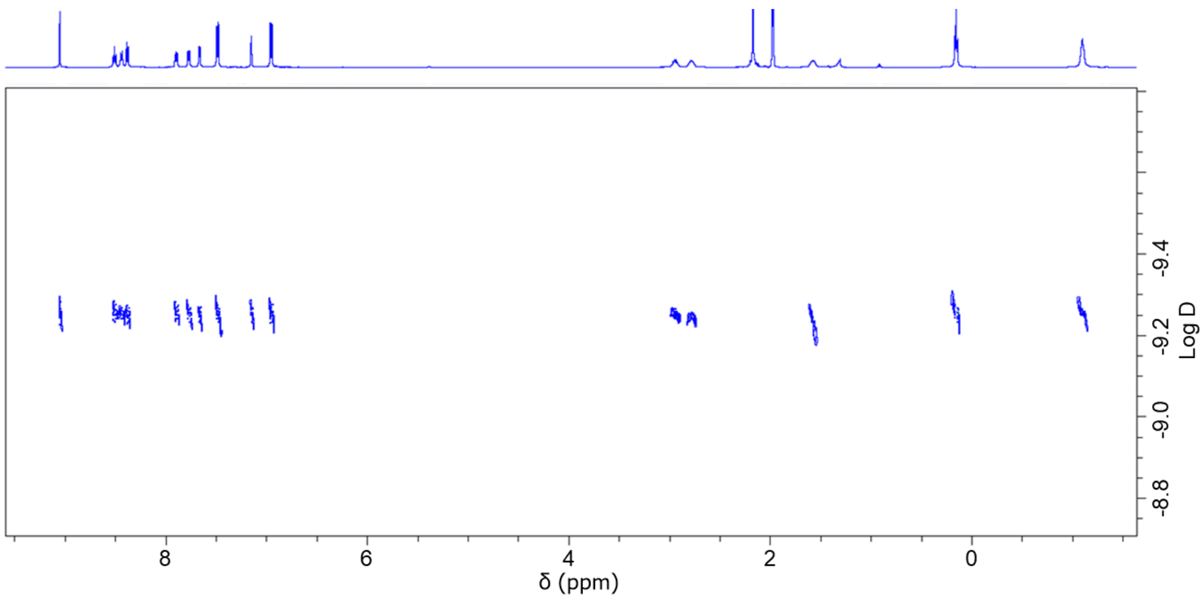

Figure S10. ^{1}H DOSY spectrum of tetrahedron **1** (500 MHz, CD_3CN, 298 K).

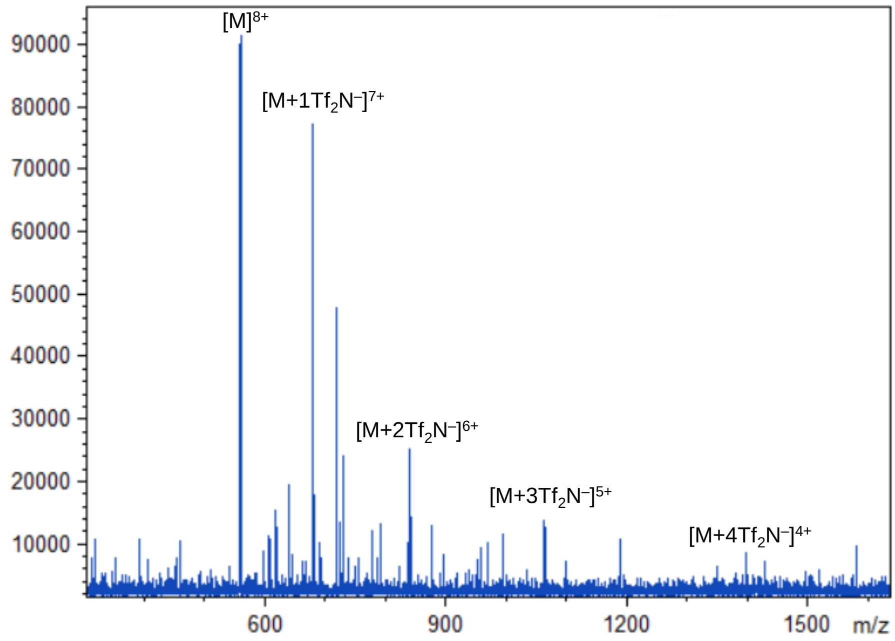

Figure S11. Low-resolution ESI-mass spectrum of tetrahedron **1**.

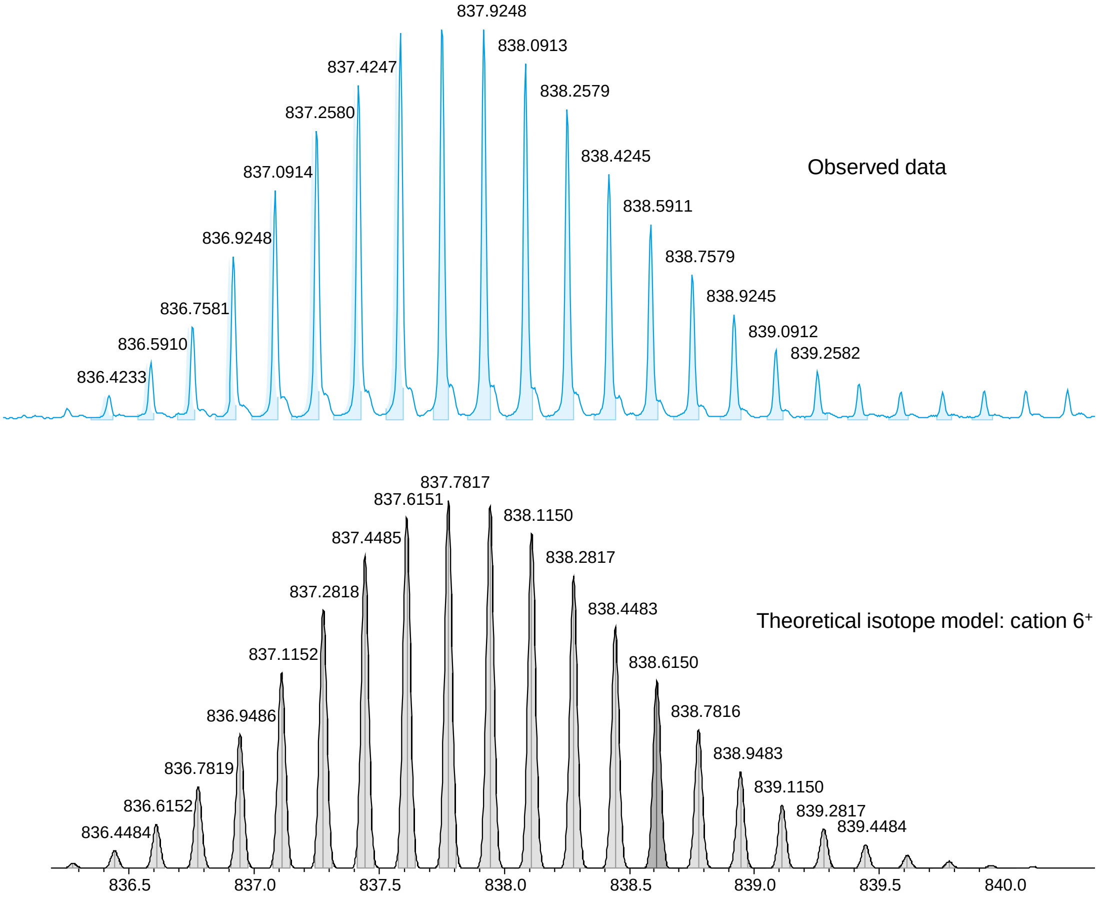

Figure S12. High-resolution ESI-mass spectrometry analysis of **1** showing the +6 peak.

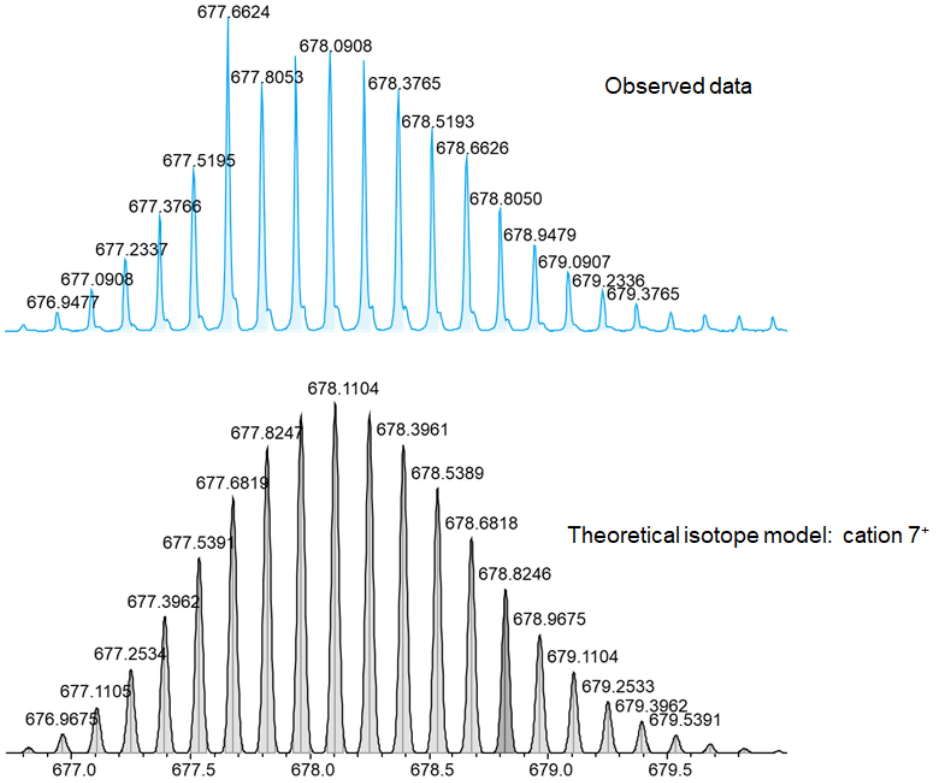

Figure S13. High-resolution ESI-mass spectrometry analysis of **1** showing the +7 peak.

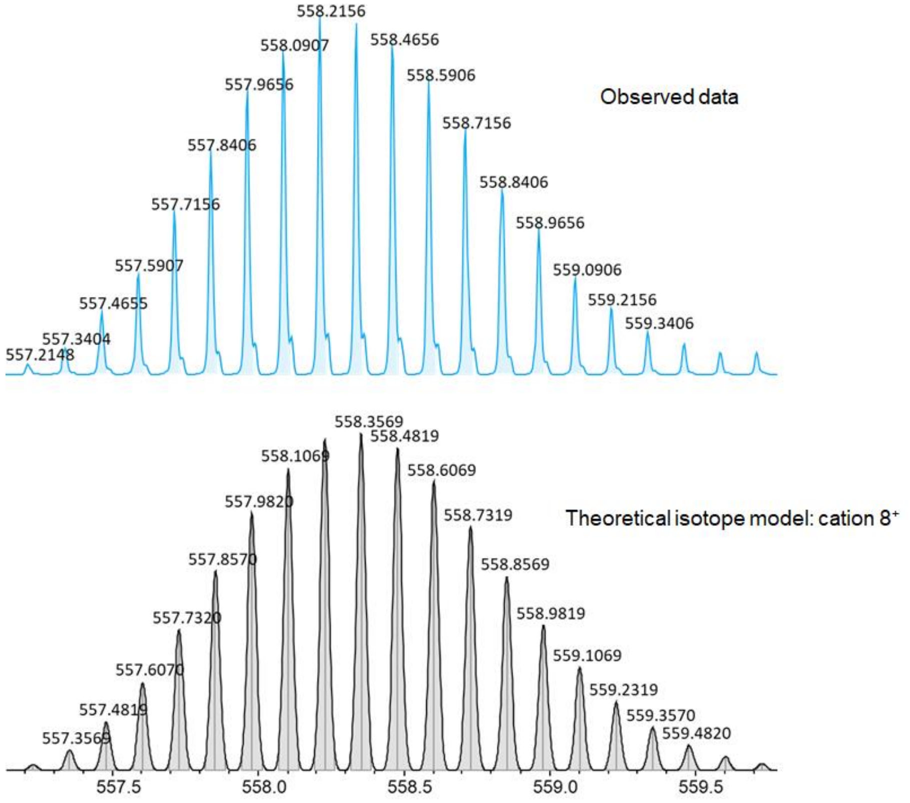

Figure S14. High-resolution ESI-mass spectrometry analysis of **1** showing the +8 peak.

3. X-ray crystallography

The crystals of **1**·8NTf$_2$·2MeCN were grown by diffusion of diethyl ether into an acetonitrile solution of the complex. Data were collected using a Bruker D8 VENTURE equipped with high-brilliance IμS Cu-Kα radiation (1.54178 Å), with ω and ψ scans at 180(2) K. Data integration and reduction were undertaken with SAINT and XPREP[2] and a multi-scan empirical absorption correction was applied to the data using SADABS.[2] Subsequent computations were carried out using the WinGX-32 graphical user interface.[3] The structure was solved by intrinsic phasing using SHELXT[4] then refined and extended with SHELXL.[5] In general, non-hydrogen atoms with occupancies greater than 0.5 were refined anisotropically. Carbon-bound hydrogen atoms were included in idealised positions and refined using a riding model. Disorder was modelled using standard crystallographic methods including restraints where necessary. Crystallographic data have been deposited with the CCDC (2060406).

The crystals employed in this study immediately lost solvent after removal from the mother liquor and rapid handling prior to flash cooling in the cryostream was required to collect data. Despite these measures and the use of a high intensity laboratory source few reflections at greater than 1.15 Å resolution were observed and the data were trimmed accordingly. Nevertheless, the quality of the data is far more than sufficient to establish the connectivity of the structure. The asymmetric unit was found to contain one complete Zn_4L_4 assembly and associated counterions and solvent molecules. Due to the limited resolution of the data, bond lengths and angles within pairs of chemically identical organic ligands were restrained to be similar to each other and thermal parameter restraints (SIMU, RIGU) were applied to all atoms except for zinc. Two of the ligand phenyl rings were modelled as disordered over two locations with bond length and angle restraints applied to achieve a reasonable model.

The anions within the structure show evidence of substantial disorder. Four triflimide anions were modelled as disordered over two locations and the occupancies of all located anions were freely refined. Substantial bond length and thermal parameter restraints were applied to facilitate a reasonable refinement of the disordered triflimide anions and most low occupancy disordered anions were modelled with isotropic thermal parameters.

Further reflecting the solvent loss and poor diffraction properties there is a significant

amount of void volume in the lattice containing smeared electron density from disordered solvent and 3 unresolved anions per Zn_4L_4 assembly (included as triflimide in the formula). Consequently the SQUEEZE[6] function of PLATON[7] was employed to remove the contribution of the electron density associated with these highly disordered anions and solvent, which gave a potential solvent accessible void of 8384 Å^3 per unit cell (a total of approximately 2140 electrons). Since the diffuse solvent molecules could not be assigned conclusively to acetonitrile or diethyl ether only those solvent molecules that could be modelled with discrete atom positions are included in the formula.

CheckCIF gives one A and two B level alerts, all resulting from the limited resolution of the data.

Table S1. Crystal data and structure refinement for $Zn_4L_4\cdot 8NTf_2\cdot 2MeCN$.

Empirical formula	C320 H270 F48 N34 O32 S16 Zn4	
Formula weight	6790.12	
Temperature	180(2) K	
Wavelength	1.54178 Å	
Crystal system	Triclinic	
Space group	P -1	
Unit cell dimensions	a = 21.6772(12) Å	α= 102.949(2)°.
b = 28.3510(16) Å	β= 95.153(3)°.	
c = 37.125(2) Å	γ = 99.154(3)°.	
Volume	21765(2) Å^3	
Z	2	
Density (calculated)	1.036 Mg/m^3	
Absorption coefficient	1.591 mm^{-1}	
F(000)	6984	
Crystal size	0.210 x 0.120 x 0.080 mm^3	
Theta range for data collection	2.266 to 42.093°.	
Index ranges	-9<=h<=18, -24<=k<=24, -32<=l<=32	
Reflections collected	67008	

Independent reflections	28897 [R(int) = 0.0483]
Completeness to theta = 42.093°	96.4 %
Absorption correction	Semi-empirical from equivalents
Max. and min. transmission	0.7488 and 0.5665
Refinement method	Full-matrix least-squares on F^2
Data / restraints / parameters	28897 / 9180 / 4033
Goodness-of-fit on F^2	1.046
Final R indices [I>2sigma(I)]	R1 = 0.1086, wR2 = 0.2803
R indices (all data)	R1 = 0.1476, wR2 = 0.3213
Largest diff. peak and hole	0.702 and -0.657 e.$Å^{-3}$

4. Volume calculations

In order to determine the available void space within **1**, a VOIDOO calculation[8] based on the crystal structure was performed. A virtual probe with a radius of 1.4 Å (set by default, water-sized) was employed, and the standard parameters tabulated below were used.[9]

Maximum number of volume-refinement cycles:	30
Minimum size of secondary grid:	3
Grid for plot files:	0.1
Primary grid spacing:	0.1
Plot grid spacing:	0.1

5. Gas binding studies of tetrahedron 1 in solution

The binding properties of tetrahedron **1** for a range of gases including methane, ethane, and ethene in solution were investigated by ^{1}H NMR experiments. After bubbling each gas into an acetonitrile solution of **1** (1.8 mM) in an NMR tube for 10 minutes at 298 K, the ^{1}H NMR spectrum was then recorded. Variable temperature (VT) ^{1}H NMR experiments were also carried out, which demonstrated the slow exchange binding of **1** at 238 K for all three gaseous guests. Based on the integrations, binding constants of **1** for all three gases were estimated to be smaller than 50 M^{-1} at 238 K.

5.1 Binding of CH_4

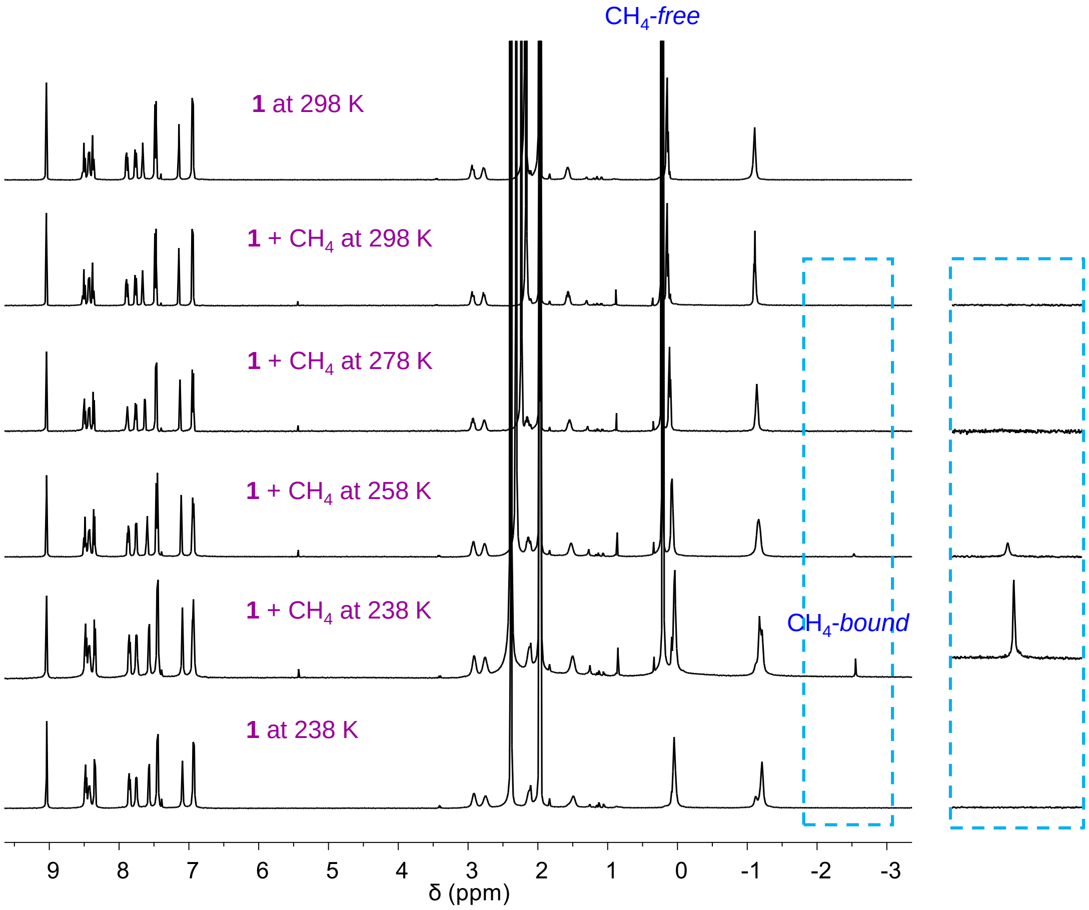

Figure S15. VT ^{1}H NMR spectra of **1** in the presence or absence of CH_4 (600 MHz, CD_3CN).

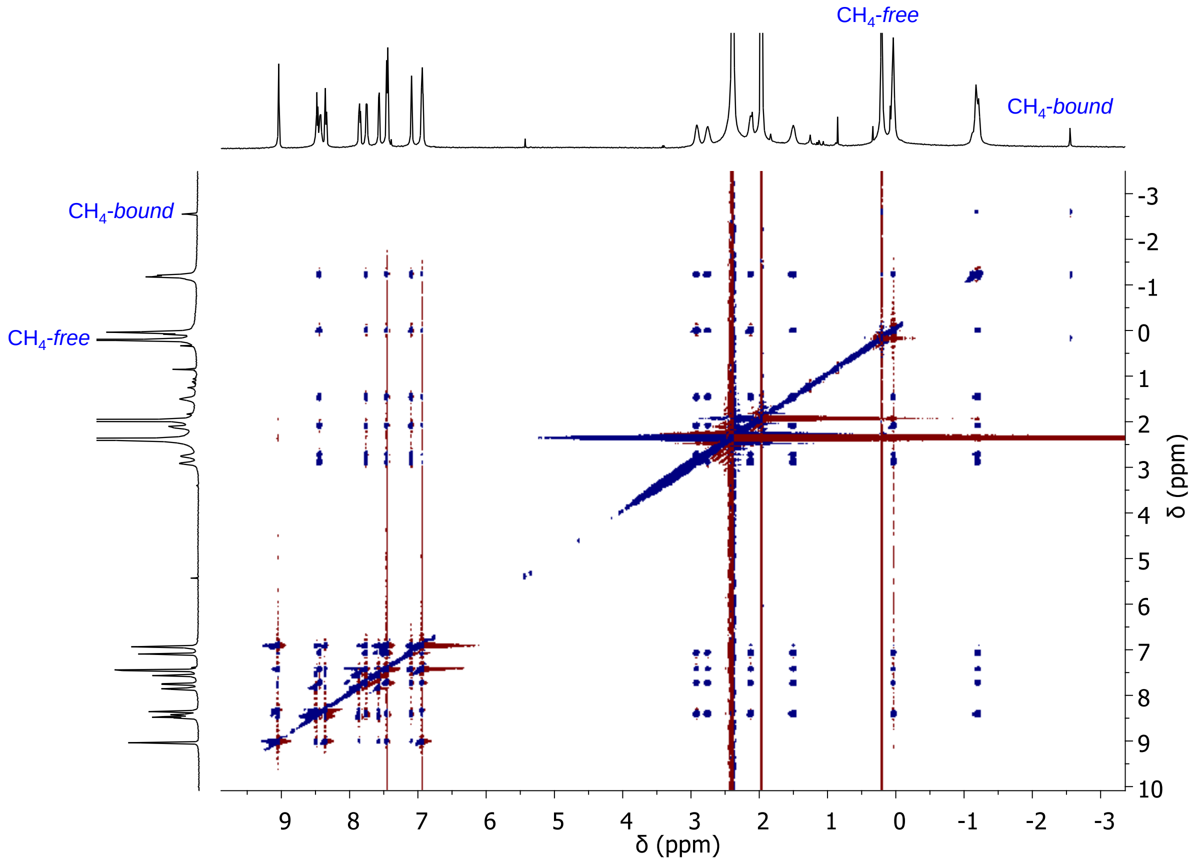

Figure S16. ^{1}H-^{1}H NOESY spectrum of tetrahedron **1** in the presence of CH_4 (600 MHz, CD_3CN, 238K).

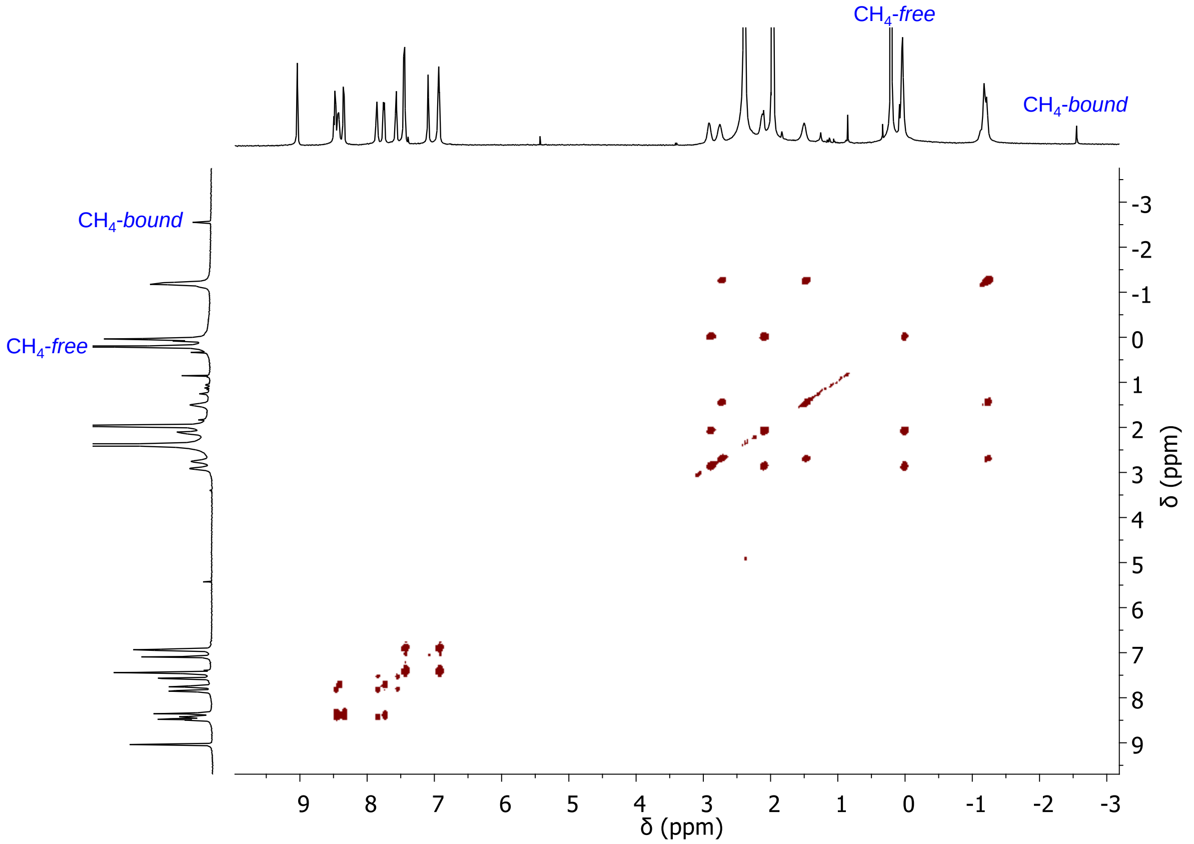

Figure S17. ^{1}H-^{1}H COSY spectrum of tetrahedron **1** in the presence of CH_4 (600 MHz, CD_3CN, 238K).

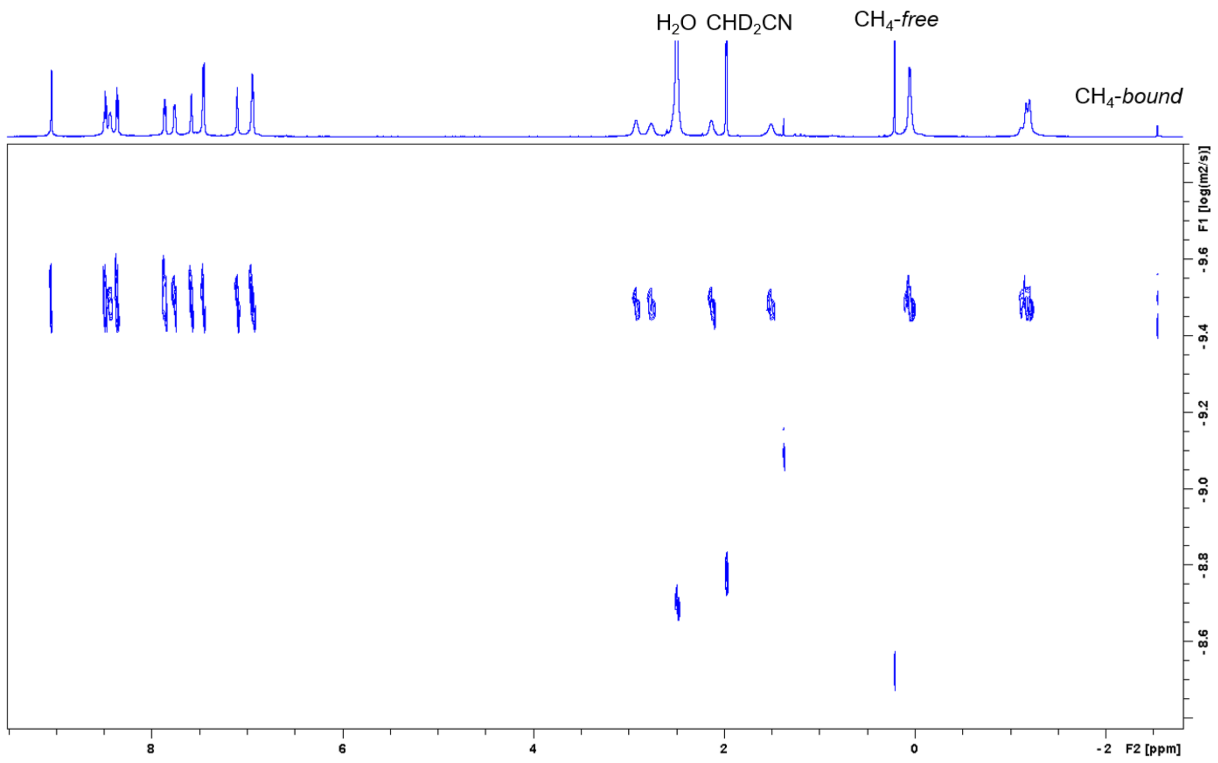

Figure S18. ^{1}H DOSY spectrum of tetrahedron **1** in the presence of CH_4 (600 MHz, CD_3CN, 238K).

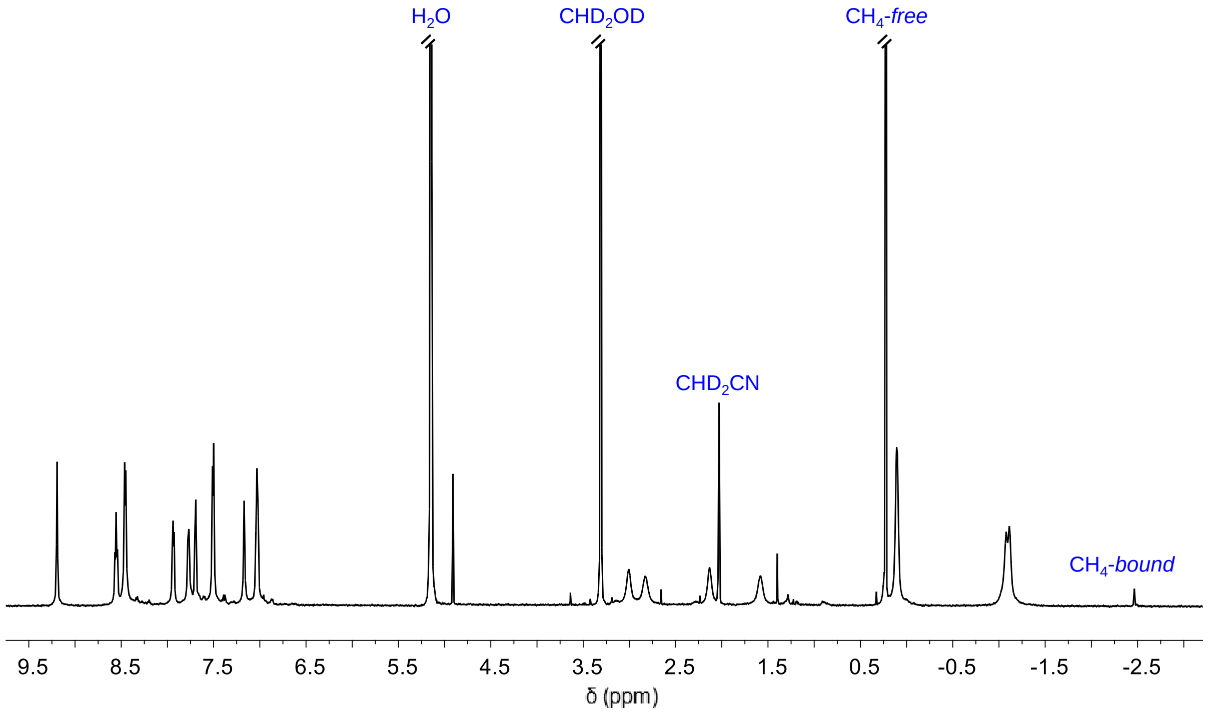

Figure S19. ^{1}H NMR spectrum of tetrahedron **1** (1 mM) in the presence of CH_4 (600 MHz, CD_3OD/CD_3CN = 4/1, 238K). The binding constant of **1** for CH_4 in a mixture of CD_3OD and CD_3CN (v/v, 4/1) is too small to be accurately determined from integrations.

5.2 Binding of C_2H_6

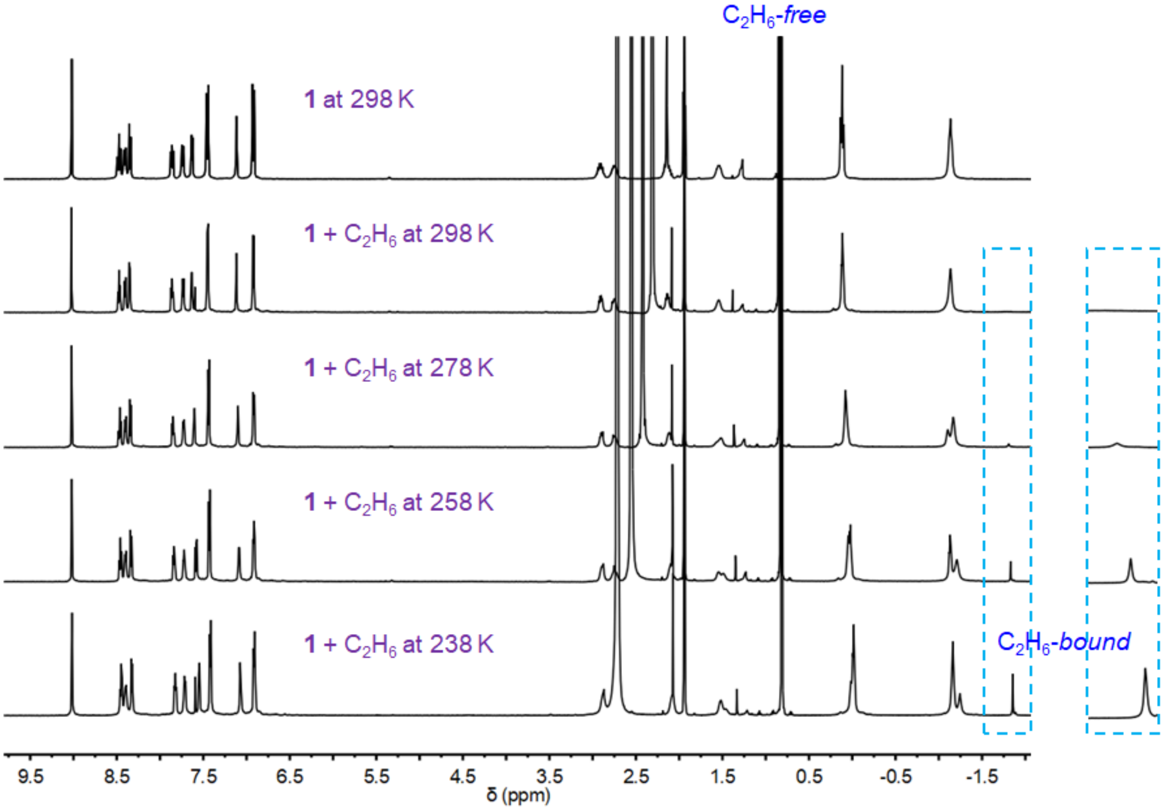

Figure S20. VT ^{1}H NMR spectra of **1** in the presence or absence of C_2H_6 (600 MHz, CD_3CN).

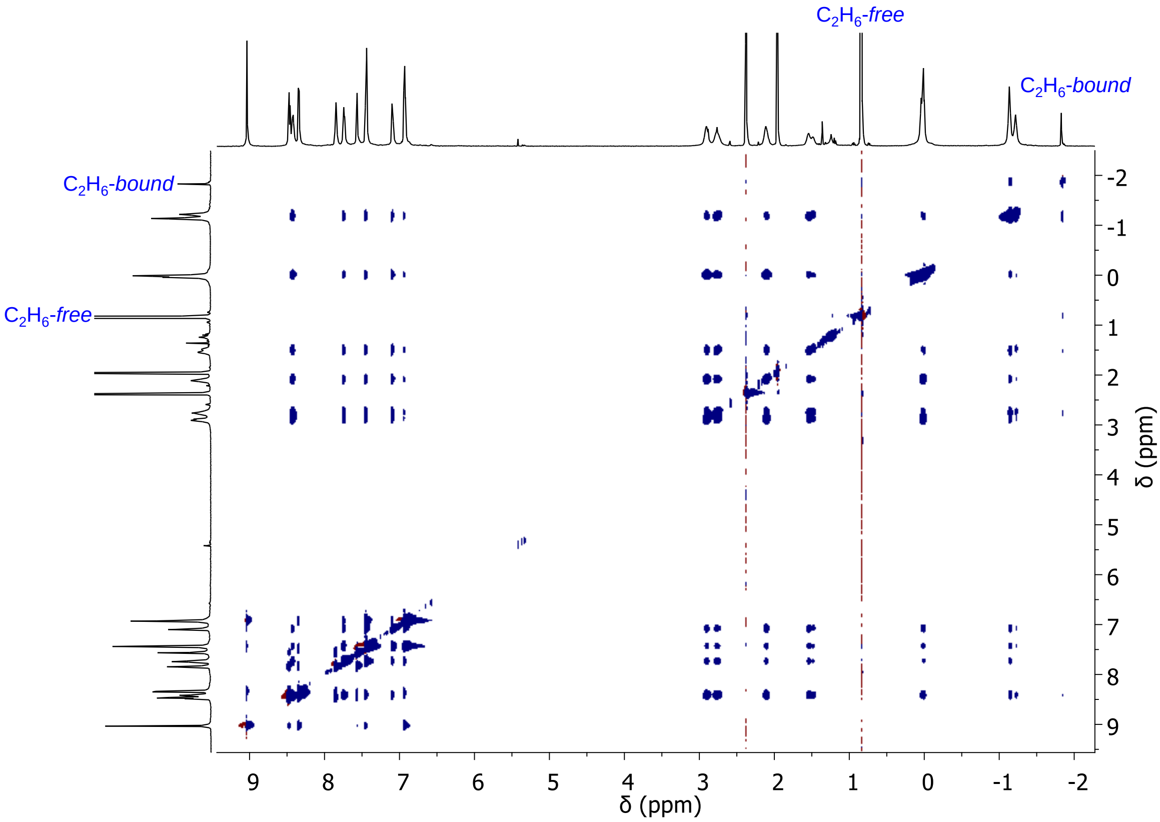

Figure S21. ^{1}H-^{1}H NOESY spectrum of tetrahedron **1** in the presence of C_2H_6 (600 MHz, CD_3CN, 238K).

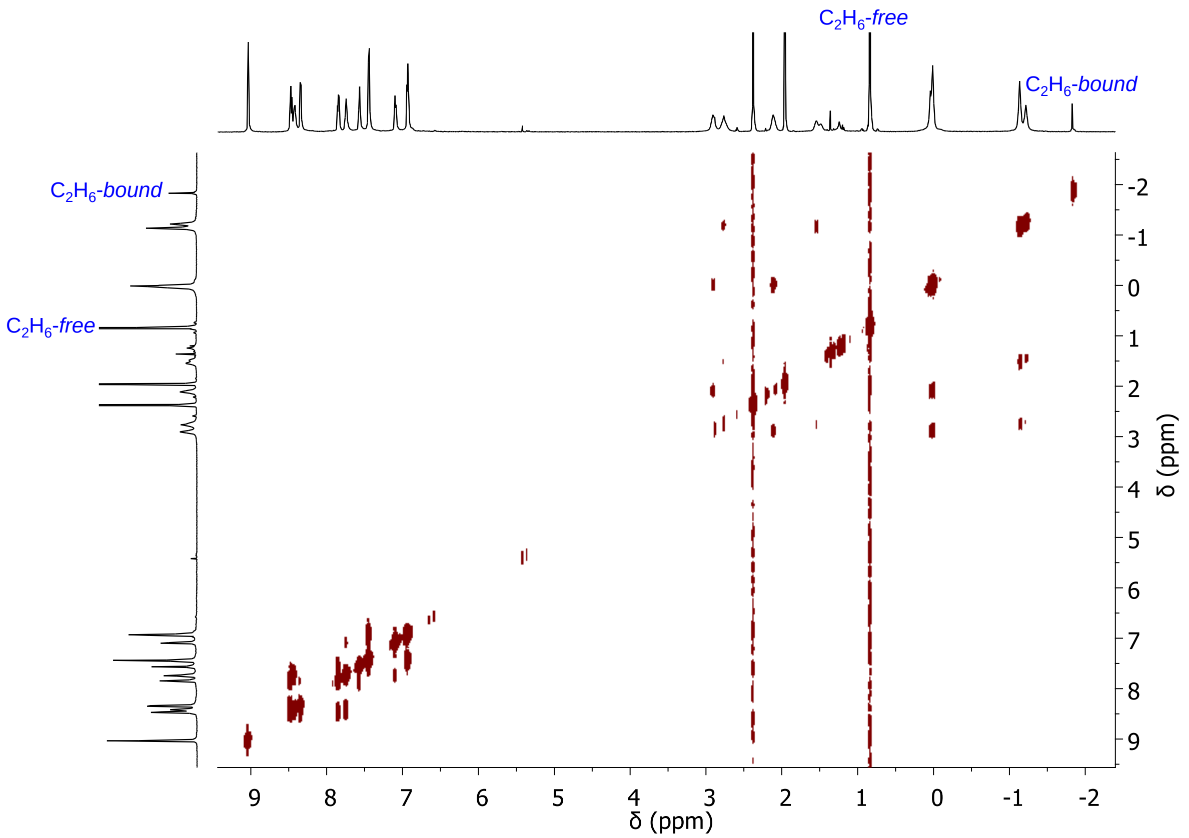

Figure S22. ^1H-^1H COSY spectrum of tetrahedron **1** in the presence of C_2H_6 (600 MHz, CD_3CN, 238K).

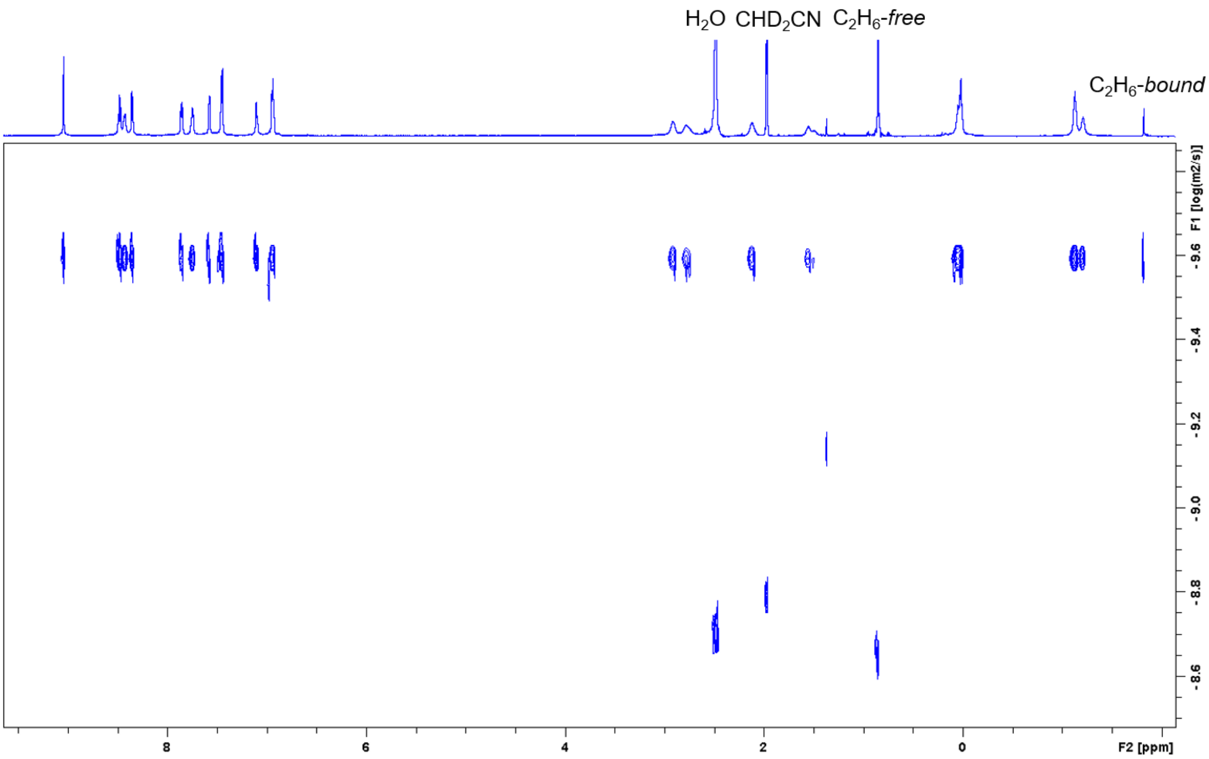

Figure S23. ^1H DOSY spectrum of tetrahedron **1** in the presence of C_2H_6 (600 MHz, CD_3CN, 238K).

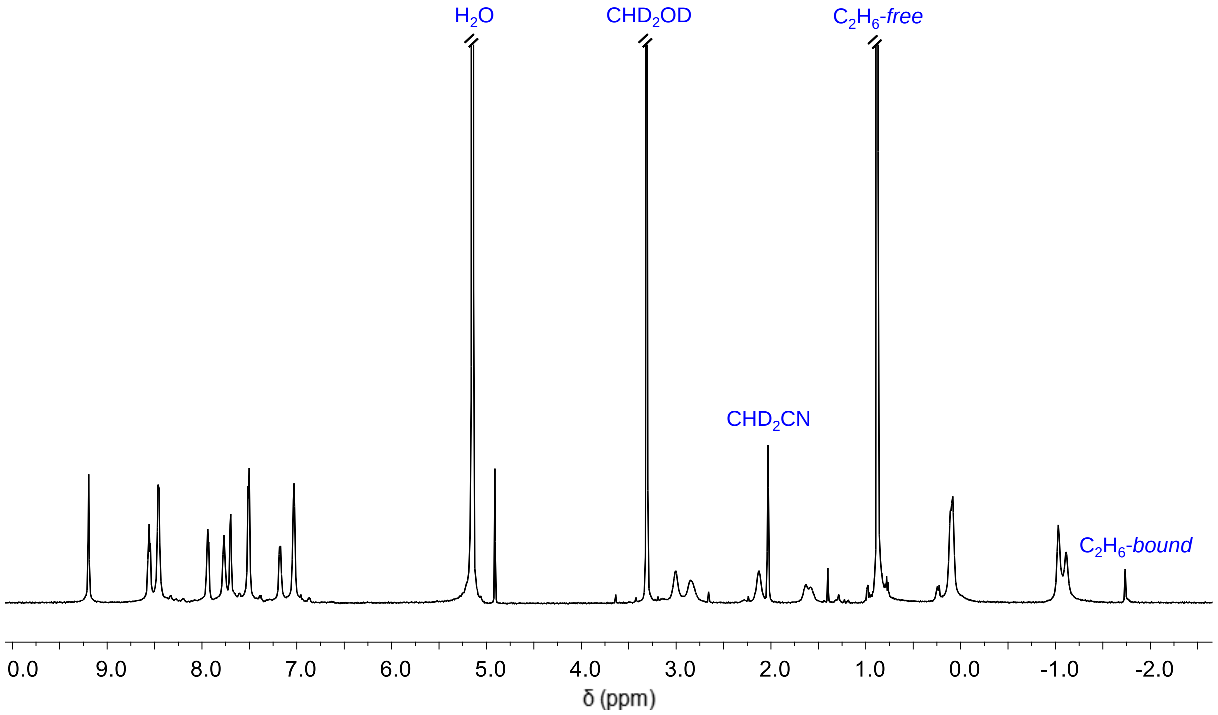

Figure S24. ^{1}H NMR spectrum of tetrahedron **1** (1 mM) in the presence of C_2H_6 (600 MHz, CD_3OD/CD_3CN = 4/1, 238K). The binding constant of **1** for C_2H_6 in a mixture of CD_3OD and CD_3CN (v/v, 4/1) is too small to be accurately determined from integrations.

5.3 Binding of C_2H_4

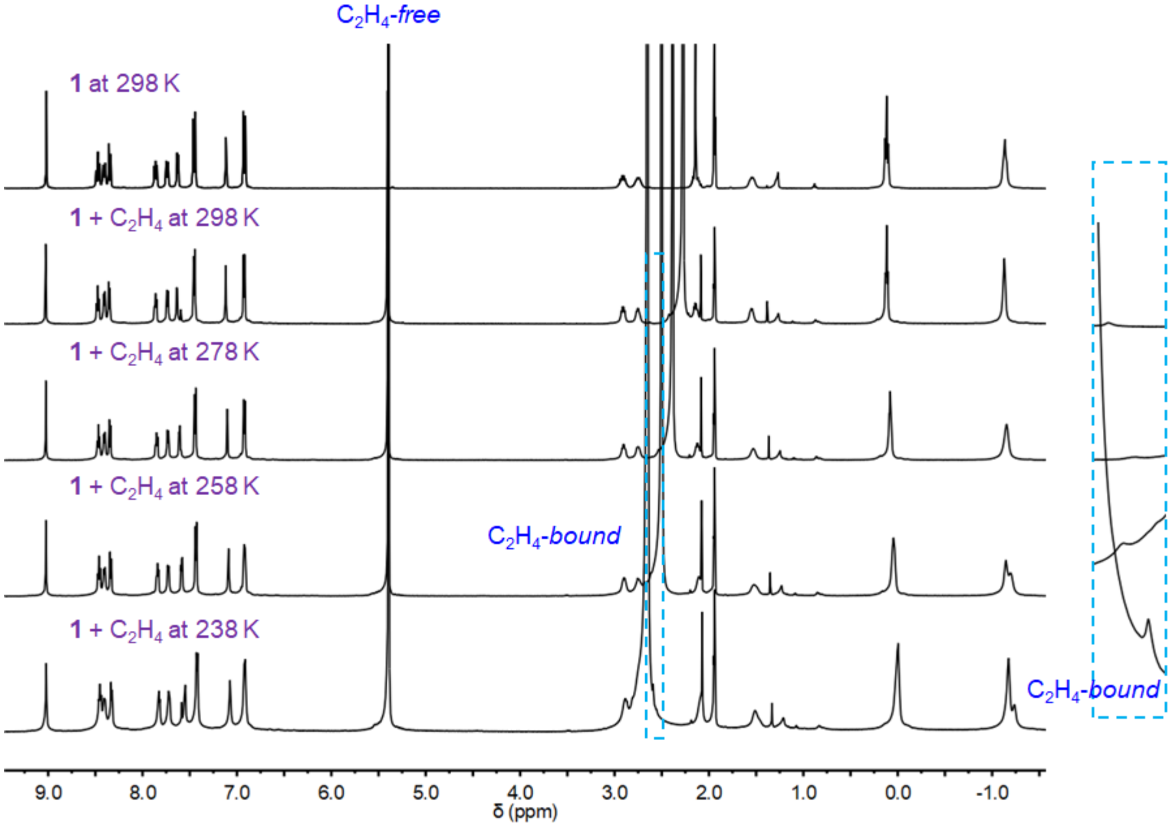

Figure S25. VT ^{1}H NMR spectra of **1** in the presence or absence of C_2H_4 (600 MHz, CD_3CN).

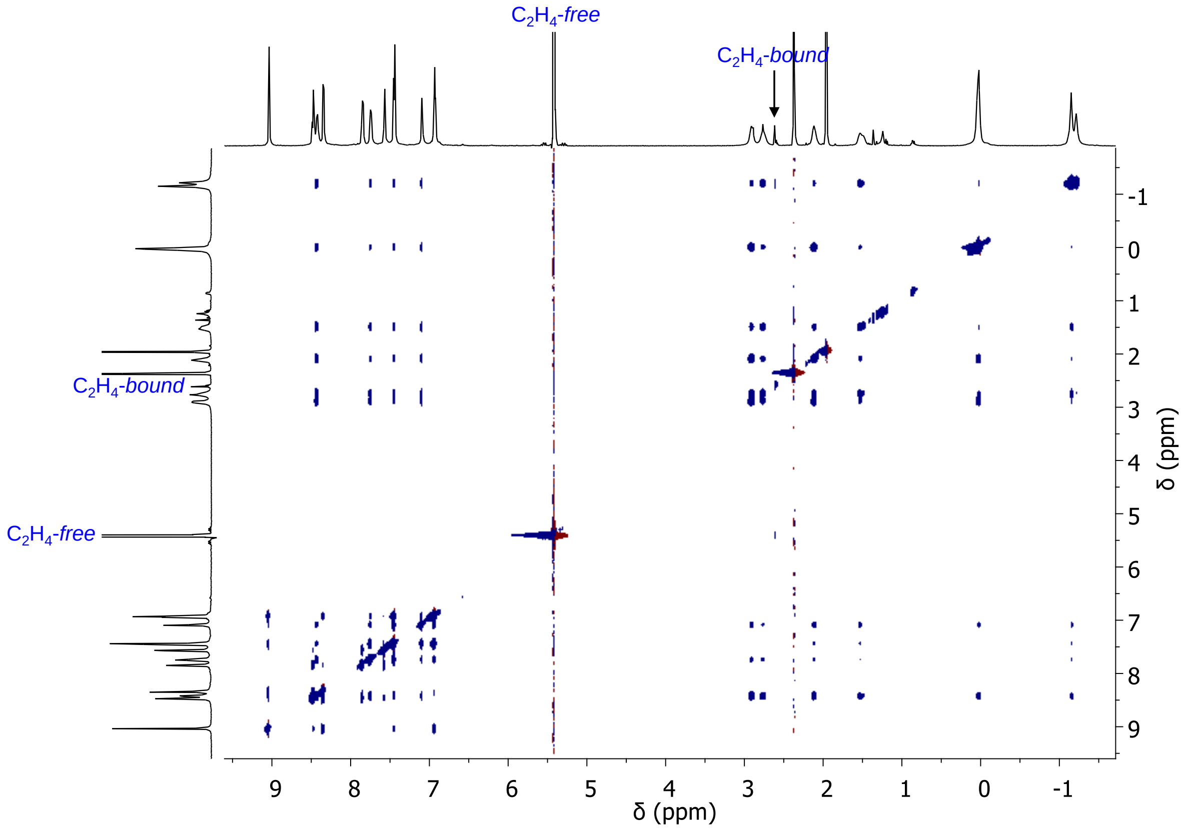

Figure S26. ^{1}H-^{1}H NOESY spectrum of tetrahedron **1** in the presence of C_2H_4 (600 MHz, CD_3CN, 238K).

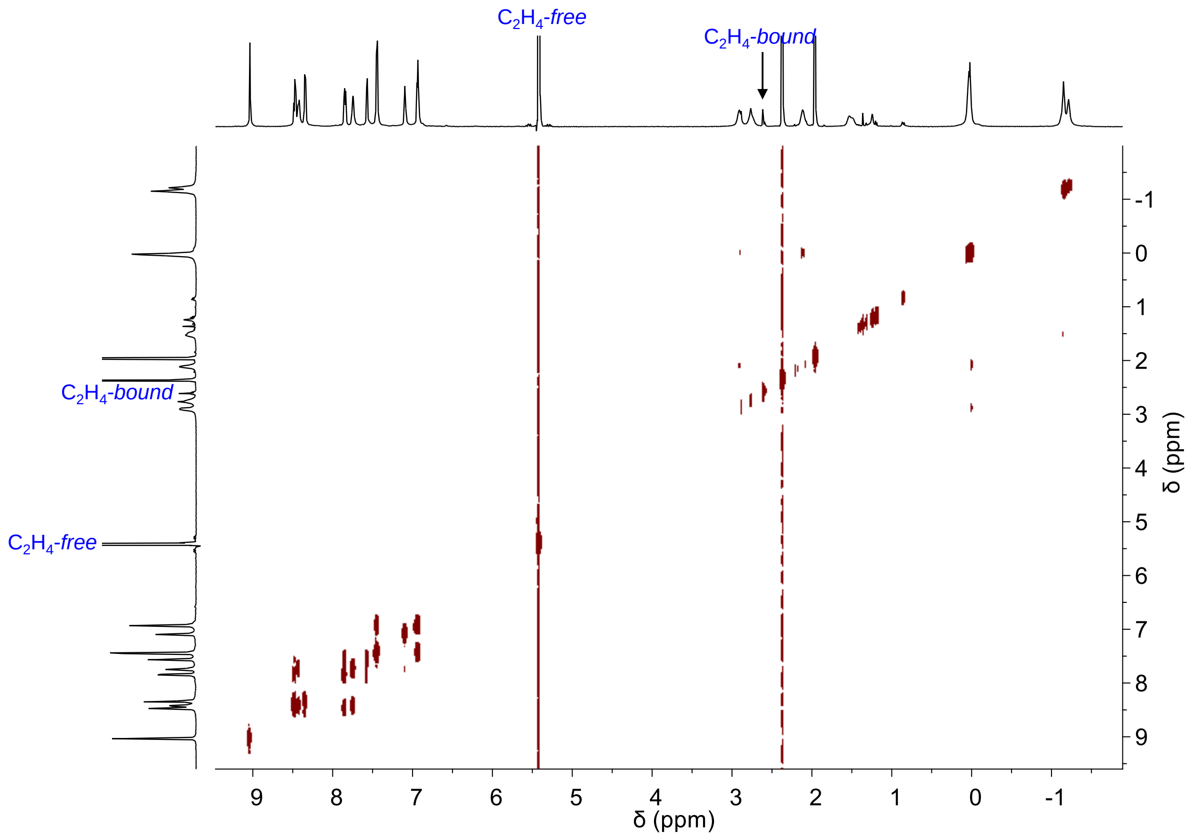

Figure S27. ^{1}H-^{1}H COSY spectrum of tetrahedron **1** in the presence of C_2H_4 (600 MHz, CD_3CN, 238K).

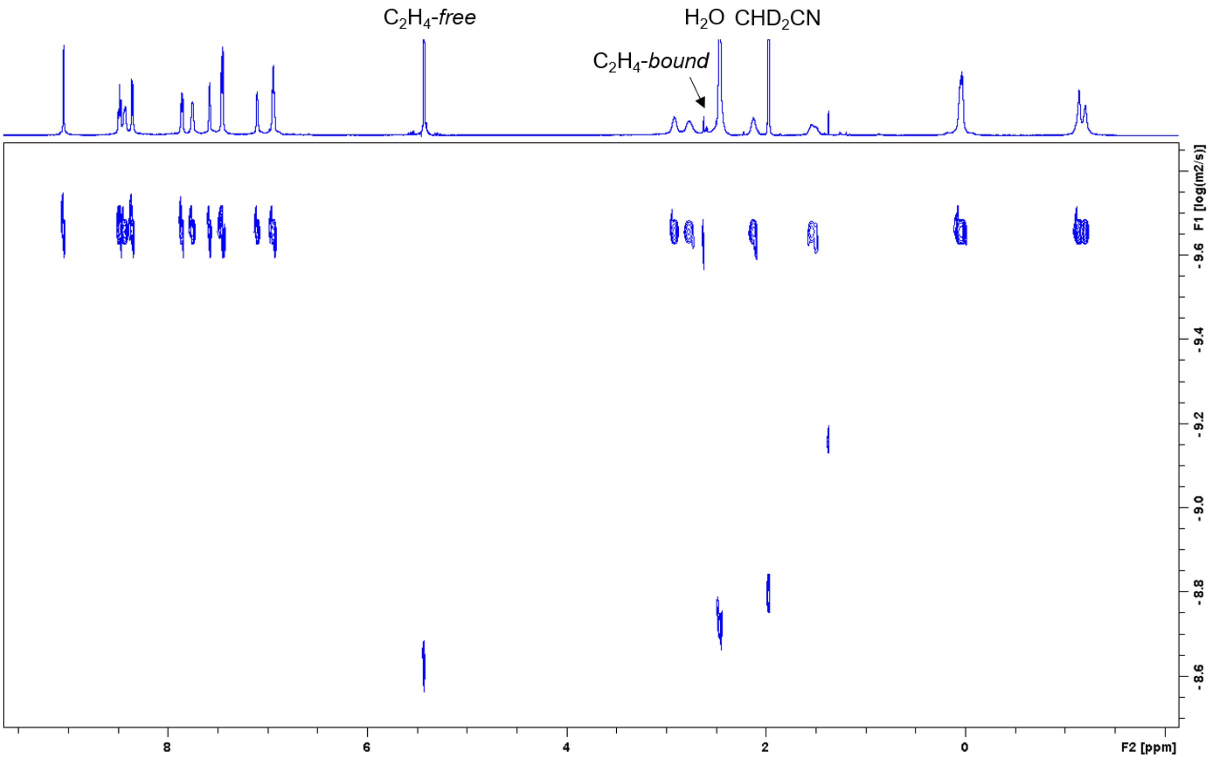

Figure S28. ^{1}H DOSY spectrum of tetrahedron **1** in the presence of C_2H_4 (600 MHz, CD_3CN, 238K).

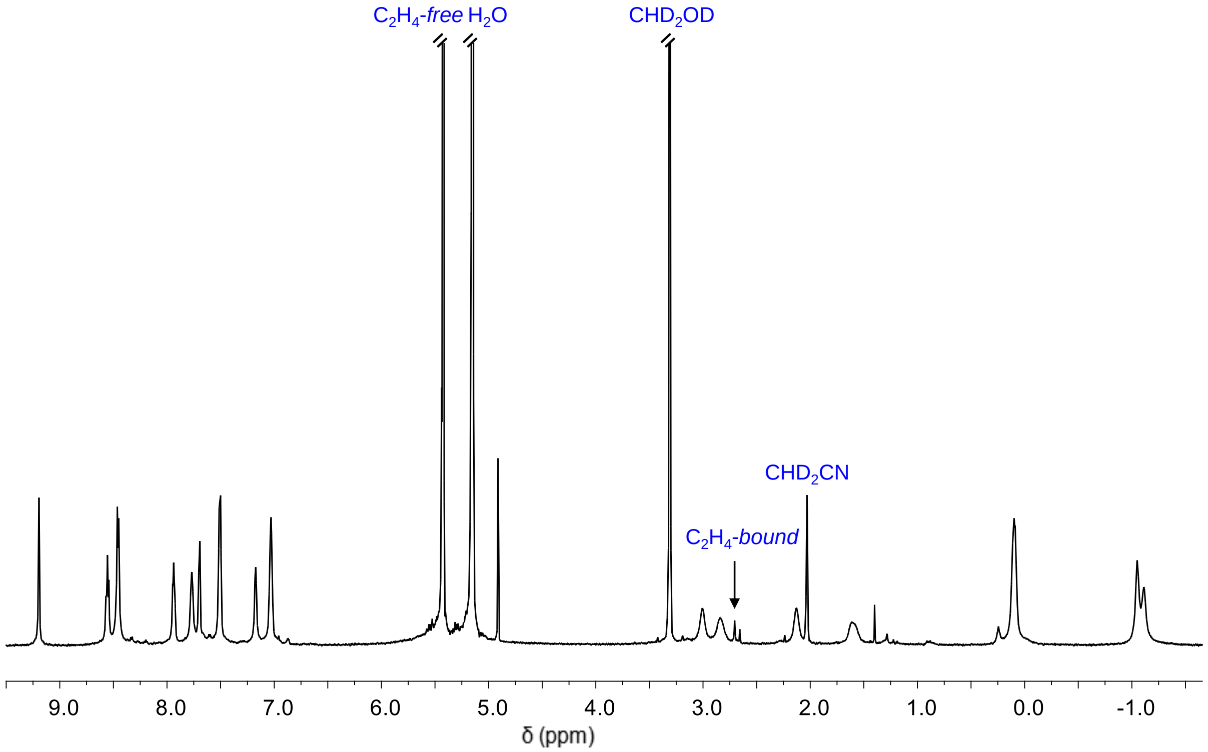

Figure S29. ^{1}H NMR spectrum of tetrahedron **1** (1 mM) in the presence of C_2H_4 (600 MHz, CD_3OD/CD_3CN = 4/1, 238K). The binding constant of **1** for C_2H_4 in a mixture of CD_3OD and CD_3CN (v/v, 4/1) is too small to be accurately determined from integrations.

5.4 Competitive gas binding studies

To determine the binding hierarchy of tetrahedron **1** in acetonitrile towards the three gases, methane, ethane, and ethene, competitive guest binding experiments were conducted. An acetonitrile solution of **1** in an NMR tube (1.8 mM) was sparged with two gases, either methane and ethane, ethane and ethene, or methane and ethene, and then the ^{1}H NMR spectrum was recorded at 238 K.

In solution, there is the following equilibrium:

$$[HG1] + [G2] \rightleftarrows [HG2] + [G1]$$

The relative binding constants (K_{rel}) could be calculated using the following equation:

$$K_{rel} = \frac{K_2}{K_1} = \frac{[HG2]}{[HG1]} \times \frac{[G1]}{[G2]}$$

Here [HG2]/[HG1] and [G1]/[G2] respectively represent the concentration ratios of the two types of bound gases and the two types of free gases, both of which can be determined from the ^{1}H signal integrations.

Based on this method, the relative binding constants of **1** for the three gases at 238 K were determined to be $K_{\mathrm{CH4}} = 1.5K_{\mathrm{C2H6}} = 1.8K_{\mathrm{C2H4}}$.

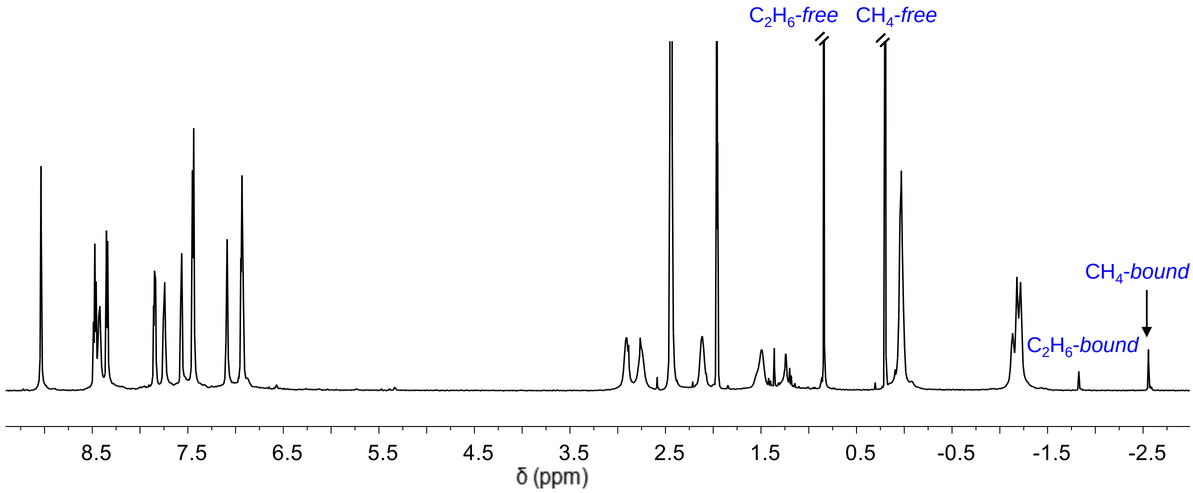

Figure S30. ^{1}H NMR spectrum (600 MHz, CD_3CN, 238 K) of tetrahedron **1** in the presence of CH_4 (**G1**) and C_2H_6 (**G2**). From the integrations, [HG2]/[HG1] = 0.33; [G1]/[G2] = 2.0; so $K_2 = 0.66K_1$ ($K_{\mathrm{CH4}} = 1.5K_{\mathrm{C2H6}}$).

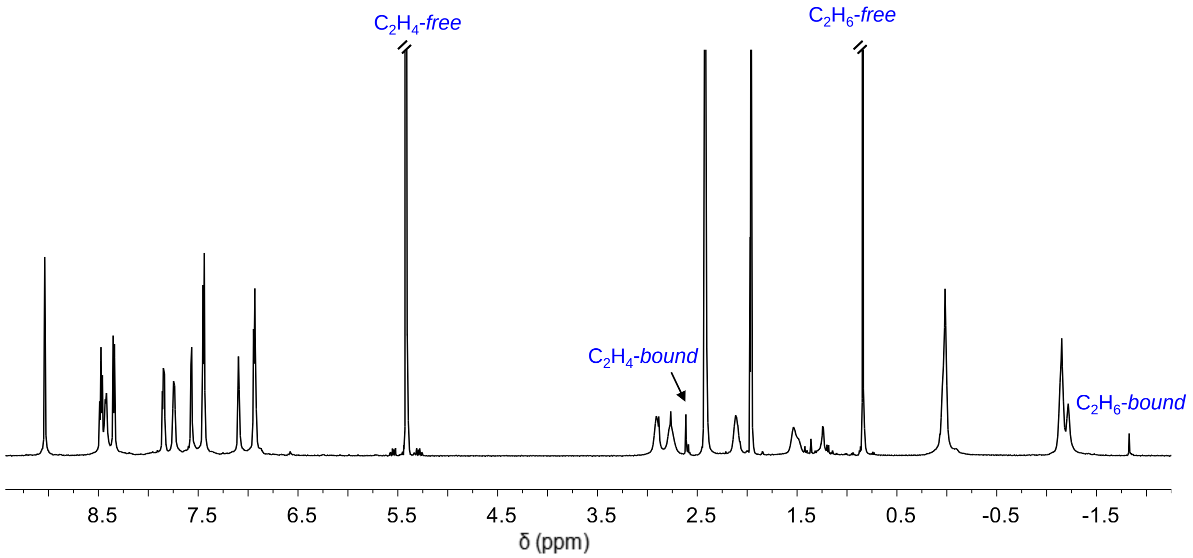

Figure S31. ^{1}H NMR spectrum (600 MHz, CD_3CN, 238 K) of tetrahedron **1** in the presence of C_2H_6 (**G1**) and C_2H_4 (**G2**). From the integrations, [HG2]/[HG1] = 3.9; [G1]/[G2] = 0.22; so $K_2 = 0.86K_1$ ($K_{C2H6} = 1.2K_{C2H4}$).

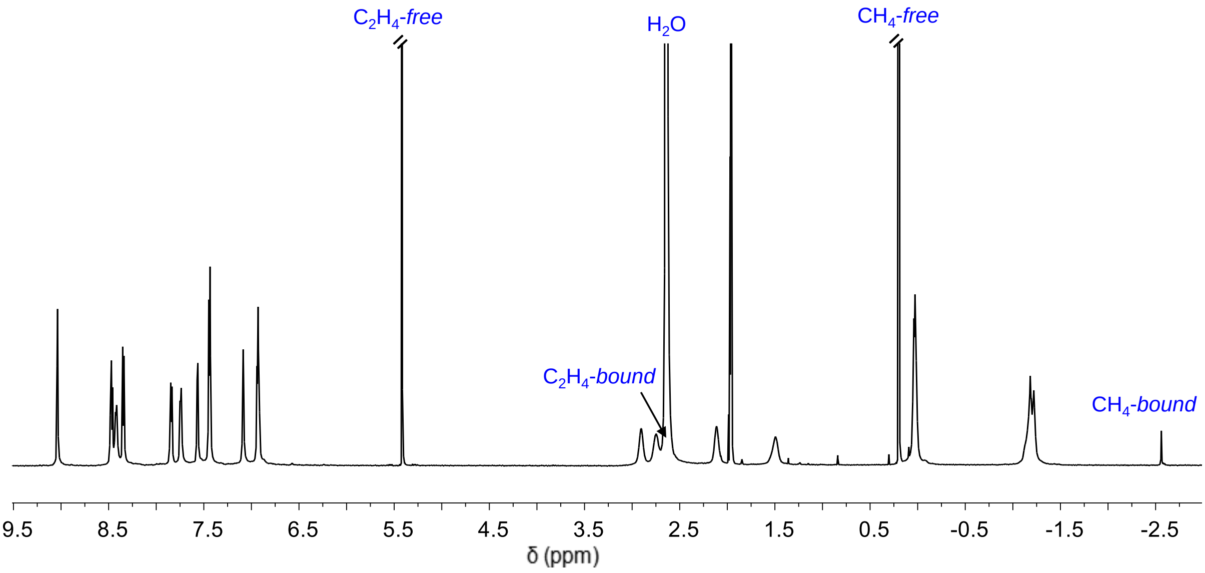

Figure S32. ^{1}H NMR spectrum (600 MHz, CD_3CN, 238 K) of tetrahedron **1** in the presence of CH_4 and C_2H_4. Due to the overlap of the bound C_2H_4 peak with the peak of H_2O, the relative binding constant between the two gases was unable to be determined from the ^{1}H NMR spectrum.

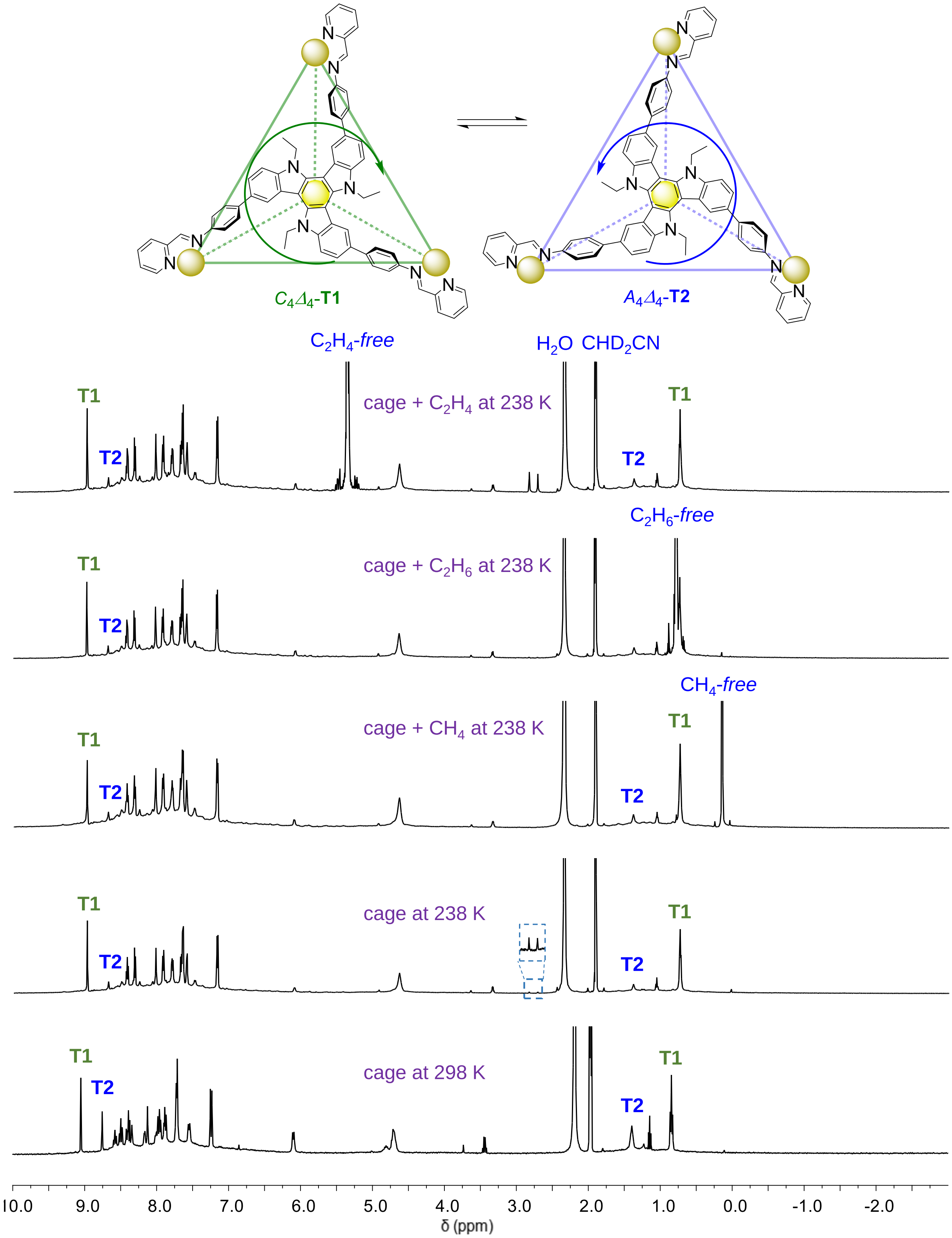

Figure S33. ^{1}H NMR spectra (600 MHz, CD_3CN) of the previously reported triazatruxene-based tetrahedra[10] containing two diastereomeric pairs of enantiomers (**T1** and **T2**) at 238 K and 298 K, and in the presence of CH_4, C_2H_6 or C_2H_4 at 238 K. No encapsulated gas peaks have been observed. The peak intensities of **T2** decrease upon lowering the temperature because of both the reduced concentration and peak broadening of **T2** at lower temperatures.

6. Characterization of amorphous solid 1

Orange cage powder **1** was obtained through direct precipitation of **1** from its acetonitrile solution after self-assembly by adding diethyl ether. The solid was dried under vacuum overnight. Prior to each measurement below, the sample was treated following the description in **Materials and instrumentation**.

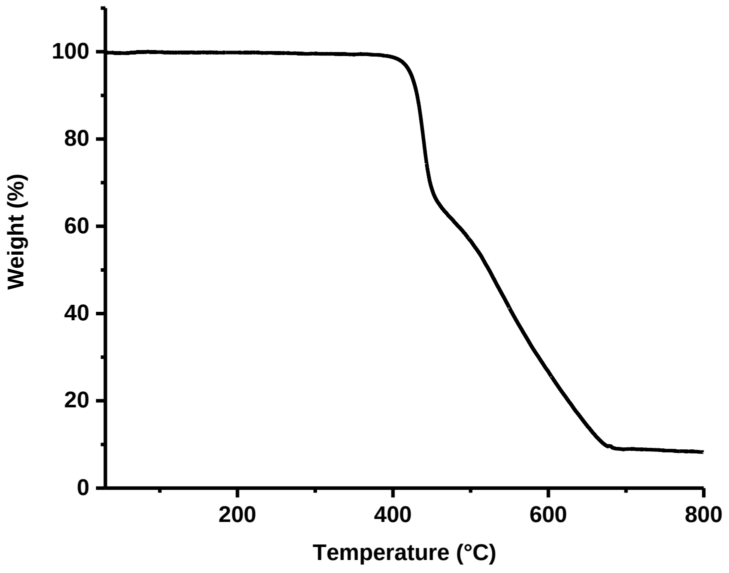

Figure S34. TGA plot of solid **1** under N_2. 5% Weight loss temperature: 423 °C.

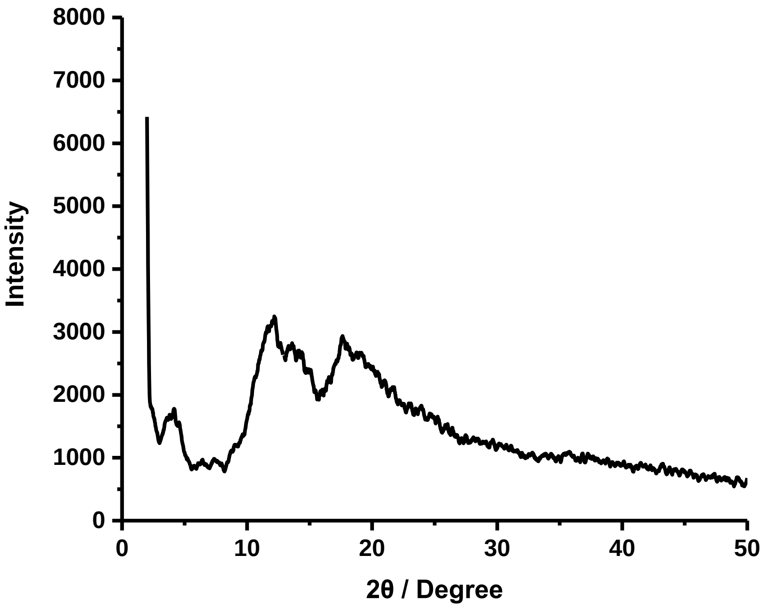

Figure S35. Experimental PXRD pattern of solid **1**.

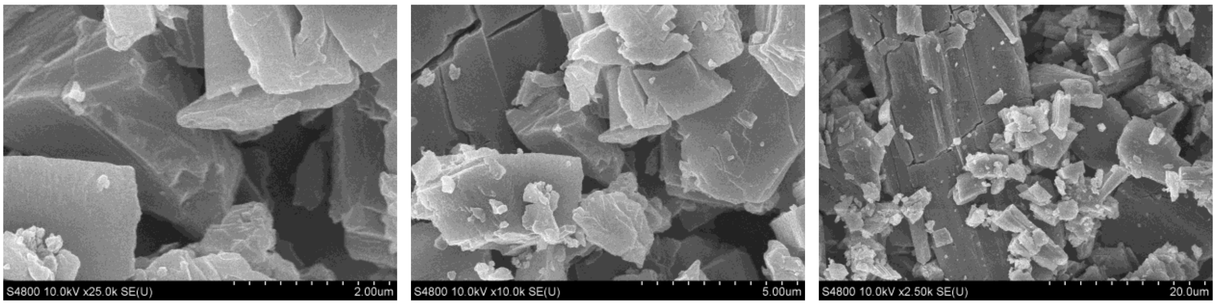

Figure S36. SEM images of solid **1**.

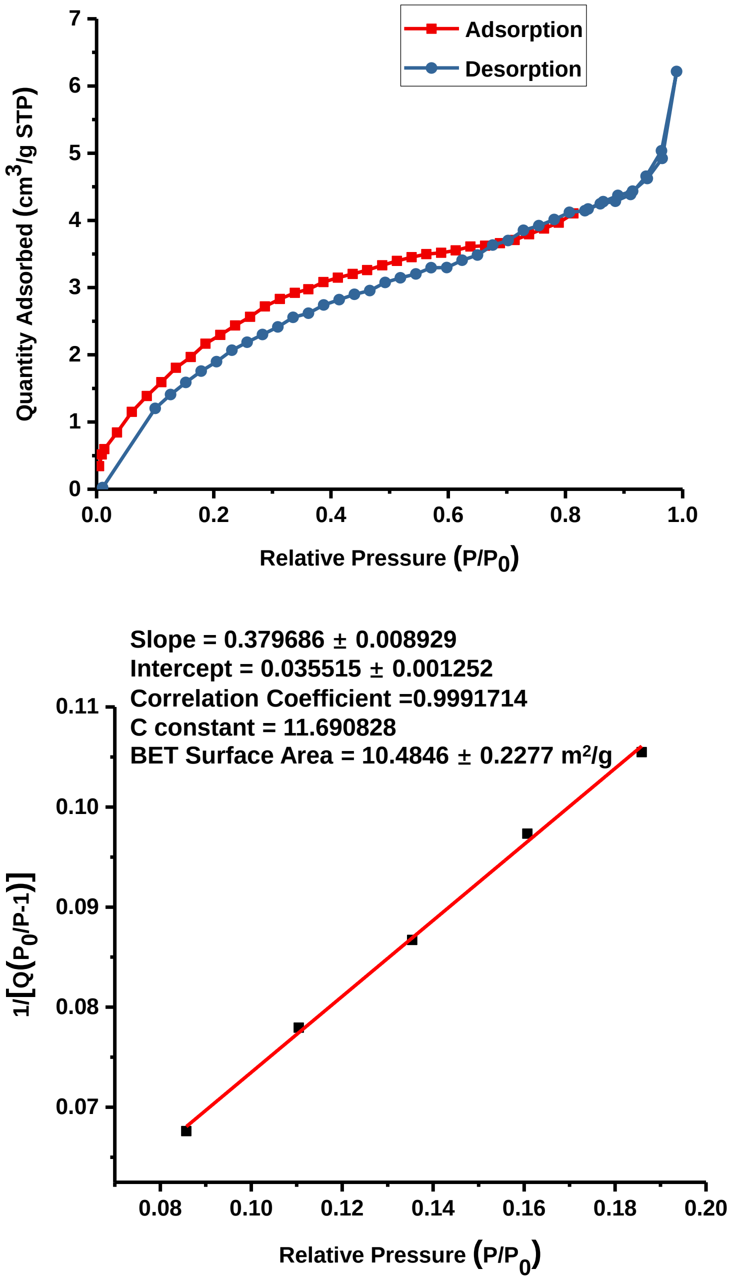

Figure S37. N_2 sorption isotherms of activated solid **1** measured at 77 K (upper) and plot of the linear region of the BET equation for solid **1** (bottom). The calculated BET surface area is 10.5 m^2/g.

7. Gas adsorption properties of solid 1

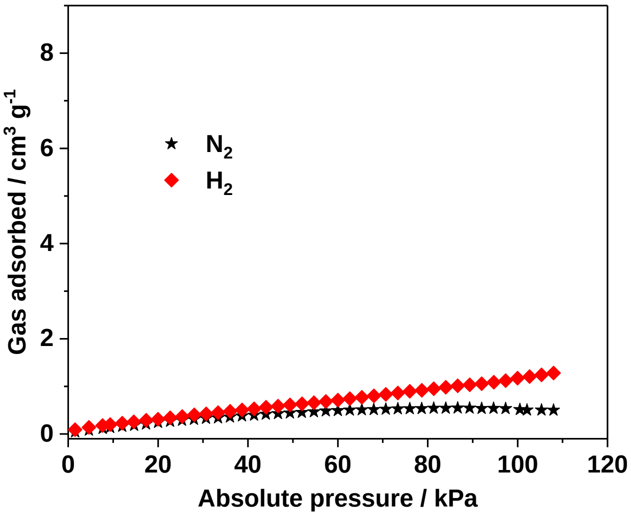

Figure S38. Gas adsorption isotherms of activated **1** for H_2 (star) and N_2 (rhombus) at 295 K.

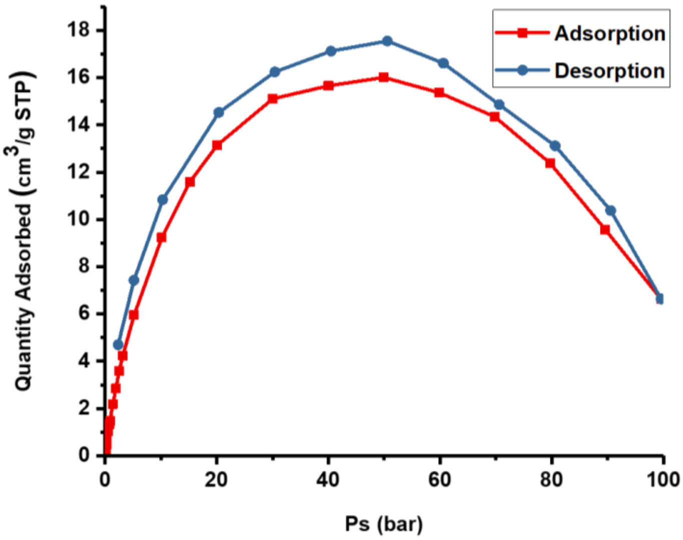

Figure S39. High-pressure excess CH_4 adsorption/desorption isotherms of activated **1** at 298 K.

It was noticed that for the high-pressure methane adsorption experiment, the adsorption isotherm displays a maximum adsorption capacity at 50 bar followed by a gradual decrease. It should be noted that the experimental quantity adsorbed corresponds to the excess adsorption (n_{ex}). The absolute adsorption (n_a) is related to the excess adsorption by the equation: $n_{ex}= n_a-\rho_g V_a$, where ρ_g is the density of the bulk gas and V_a is the exact volume occupied by the adsorbed phase.[11] The absolute adsorption n_a increases with pressure, and then saturates. However, the value of $\rho_g V_a$ continually increases with pressure according to the real gas law. Thus, n_{ex} reaches a maximum adsorption amount upon the saturation of n_a and then gradually decreases.

8. References

[1] X. Wang, Y. Wang, H. Yang, H. Fang, R. Chen, Y. Sun, N. Zheng, K. Tan, X. Lu, Z. Tian, X. Cao, *Nat. Commun.* **2016**, *7*, 12469.

[2] Bruker-Nonius, *APEX, SAINT and XPREP*, Bruker AXS Inc., Madison, Wisconsin, USA, **2013**.

[3] L. Farrugia, *J. Appl. Crystallogr.* **2012**, *45*, 849-854.

[4] G. Sheldrick, *Acta. Cryst.* **2015**, *A71*, 3-8.

[5] G. M. Sheldrick, *Acta. Cryst.* **2015**, *C71*, 3-8.

[6] P. van der Sluis, A. L. Spek, *Acta Cryst.* **1990**, *A46*, 194-201.

[7] A. L. Spek, *PLATON: A Multipurpose Crystallographic Tool*, Utrecht University, Utrecht, The Netherlands, **2008**.

[8] G. J. Kleywegt, T. A. Jones, *Acta Cryst.* **1994**, *D50*, 178-185.

[9] Y. R. Hristova, M. M. J. Smulders, J. K. Clegg, B. Breiner, J. R. Nitschke, *Chem. Sci.* **2011**, *2*, 638-641.

[10] D. Zhang, T. K. Ronson, S. Guryel, J. D. Thoburn, D. J. Wales, J. R. Nitschke, *J. Am. Chem. Soc.* **2019**, *141*, 14534-14538.

[11] M.-A. Richard, D. Cossement, P.-A. Chandonia, R. Chahine, D. Mori, K. Hirose, *AIChE J.* **2009**, *55*, 2985-2996.
